# Light-driven CO_2_ reduction with substituted imidazole-pyridine Re catalysts favoring formic acid production†

**DOI:** 10.1039/d5ra01561h

**Published:** 2025-04-22

**Authors:** Ryan Chafin, Majharul Islam Sujan, Sean Parkin, Jonah W. Jurss, Aron J. Huckaba

**Affiliations:** a Department of Chemistry, University of Kentucky Lexington KY 40506 USA aron.huckaba@uky.edu; b Department of Chemistry and Biochemistry, University of Mississippi Mississippi 38677 USA

## Abstract

Removing carbon dioxide from the atmosphere is an attractive way to mitigate the greenhouse gas effect that contributes to climate change. A series of donor-pi (D-π), acceptor-pi (A-π), and π Re(i) pyridyl imidazole complexes have been synthesized and examined under photocatalytic conditions for the photocatalytic reduction of CO_2_. The catalytic activity of the complexes was further supported by cyclic voltammetry through the presence of a catalytic current under CO_2_ atmosphere. The D-π, π, and A-π complexes were studied to elucidate the effects of incorporating conjugated electron donating *vs.* withdrawing groups on the catalytic rates and product selectivity. The synthesized complexes were compared with Re(bpy)(CO)_3_Br (where bpy is 2,2′-bipyridine), the benchmark catalyst for this transformation. Remarkably, the complex with A-π pendant (RC4) outperformed the π (RC2–3) and D-π (RC5) complexes for the production of formic acid (HCO_2_H) in the presence of photosensitizer [Ru(bpy)_3_]^2+^ and sacrificial electron donor BIH (1,3-dimethyl-2-phenyl-2,3-dihydro-1*H*-benzo[*d*]-imidazoline). Among the investigated catalysts, RC4 with the A-π pendant showed the highest turnover number (TON) value of 844 for HCO_2_H production with 86% carbon selectivity. In stark contrast to the imidazole-pyridine based catalysts reported here that favor formic acid as a product, Re(bpy)(CO)_3_Br generated no formic acid under the same conditions. The imidazole-pyridine complexes also function as catalysts for CO_2_ reduction without an added photosensitizer, however, the TON values under self-sensitized conditions are poor.

## Abstract 2

Novel imidazole-pyridine photocatalysts favoring selectivity for formic acid production.

## Graphical abstract

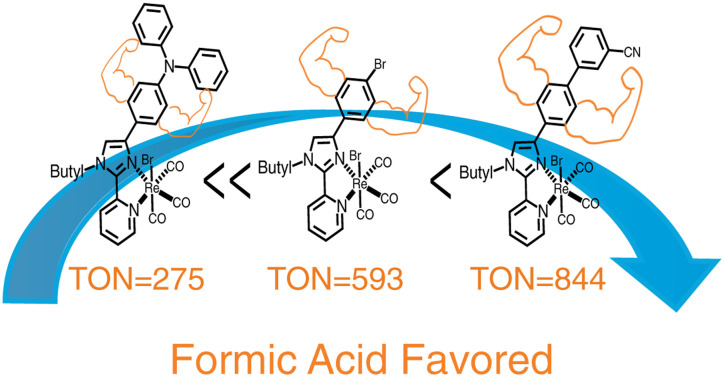

## Introduction

Currently, CO_2_ is the largest greenhouse gas emitted in the world.^1^ There is a considerable effort to convert this relatively untapped waste product into renewable fuels. While the thermodynamic reduction potentials of transforming CO_2_ to various products (CO, formic acid, methanol, methane, *etc.*) can be modest depending on the conditions,^2^ the kinetics are slow and a catalyst is required to lower activation barriers and direct selectivity for the desired product.^3^ Ideally, the energy required for catalysis is obtained directly from the sun or indirectly using solar photovoltaics to drive the electrochemistry. One reasonable goal for CO_2_ valorization is to selectively reduce CO_2_ to formic acid (HCO_2_H), which can be used directly in formic acid fuel cells^4^ or as a safe and easily transported H_2_ storage material.^5,6^ We note that CO is also a valuable product that can be transformed into hydrocarbon fuels *via* the Fischer–Tropsch process.^7^

The most well-studied mononuclear photocatalysts are derived from Lehn's original Re(bpy)(CO)_3_X (X = Cl, Br) catalyst,^8–10^ which has been modified extensively with the goal to improve the catalytic performance.^11–14^ Modification of the bidentate ligand has included introducing redox sites,^15^ ancillary Lewis acids,^16^ and other moieties to the second coordination sphere^17^ (proton relays,^18^ charged groups,^19^ hydrogen bond donors^20,21^) to facilitate proton transfer events or stabilize intermediates,^8^ change the local pH,^22^ or increase the onset of visible light absorption.^23^ Recently, Liang-Nian and coworkers reported an approach to increase the efficiency of the Re(bpy)-based photocatalyst by introducing chromophores onto the bipyridyl ligand, which yielded TONs as high as 1323 and *Φ*_CO_ up to 55%.^24^ Another approach by Jurss and coworkers studied the effect of pendant light absorbing anthracene chromophores in mono- and di-nuclear anthracenyl Re(bpy) catalysts and found that especially in di-nuclear catalysts the ligand-based triplet state was highly influential.^25^ With these two works as a conceptual backdrop, we seek to improve the overall light absorption and increase the activity for Re-based photocatalysts. We hypothesized that incorporating a ligand design with an enhanced charge transfer absorption, either in a donor–acceptor (D–A) or acceptor–donor (A–D) configuration would provide a strongly red-shifted charge transfer absorption.^26^

**Fig. 1:**
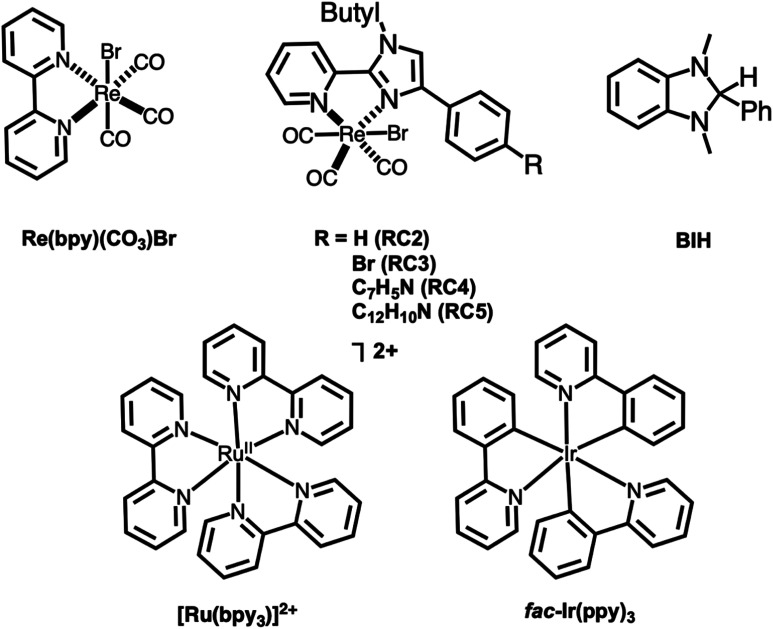
Structures of complexes studied herein. The photosensitizers used and sacrificial electron donor (BIH) are also shown.

Herein, we report the synthesis, characterization, and catalytic activity of a series of novel Re(i) imidazolylpyridine complexes (Fig. 1). We were able to confirm two of the metal complex structures through single crystal X-ray diffraction. The electrocatalytic and photocatalytic trials indicated that the complexes are able to reduce CO_2_ in sensitized photocatalytic reactions, and were modestly selective for HCO_2_H, which is rare for this class of Re photocatalysts.^27–29^

## Results and discussion

### Catalyst synthesis

**Scheme 1:**
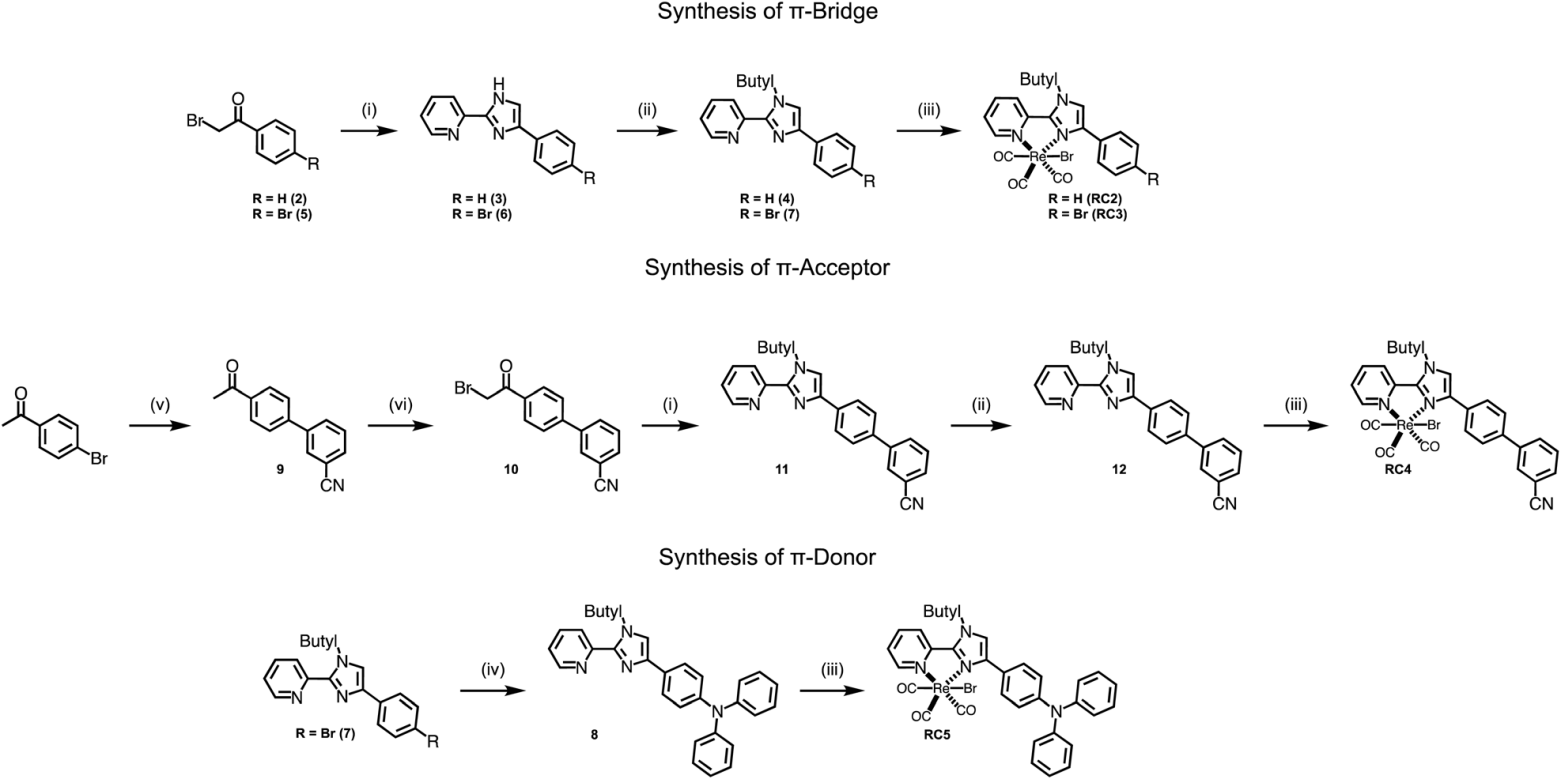
Synthetic steps to prepare complexes RC2–RC5. Reaction conditions: (i) KHCO_3_, H_2_O, THF, amino(pyridin-2-yl)methaniminium chloride, reflux. Yields: 3: 70%; 6: 70%; 11: 75%. (ii) 1-Bromobutane, DMF, NaH, 80 °C. Yields: 4: 60%; 7: 77%; 12: 30%. (iii) Re(CO)_5_Br, toluene, reflux. Yields: 13: 55%; 14: 57%; 15: 23%; 16: 60%. (iv) Diphenyl amine, toluene, KO*t*Bu, Pd(dba)_2_, tri-*tert*-butylphosphonium tetrafluoroborate, reflux, 76%. (v) 3-Cyanophenylboronic acid, Na_2_CO_3_, Pd(PPh_3_)_4_, 5 : 1 dioxane : water, 72%. (vi) *p*-TsOH, NBS, MeCN, reflux, 60%.

The synthesis of complexes RC2 and RC3 begin with an imidazole forming condensation between the alpha-brominated ketones 2, 5, or 10 and 2-amidinylpyridine to afford 3, 6, and 11 (Scheme 1).^30^ Imidazole alkylation with 1-bromobutane using NaH afforded 4, 7, and 12 in moderate yields. Alkylation of the imidazole unit was crucial to the success of the synthesis as the non-alkylated pyridyl-imidazole ligand was observed to chelate the Re metal center using either of the imidazole N atoms due to tautomerization. This led to mixtures of the two possible isomers that were not separable.^31^ Upon alkylation, however, only one isomer was observed to form, and Nuclear Overhauser Effect (NOESY) spectroscopy was performed to confirm which isomer was present after purification (Fig. S4†). Refluxing Re(CO)_5_Br in toluene with 4, 7, or 12 afforded catalysts RC2, RC3, or RC4. Catalyst RC5 was afforded with relative ease by Buchwald-coupling of diphenylamine with 7, followed by metalation with Re(CO)_5_Br in refluxing toluene. Suzuki-coupling of 3-cyanophenylboronic acid and 4-bromoacetophenone was performed to afford 9 which was then brominated at the ketone α carbon with NBS and *p*-toluenesulfonic acid to give 10. Detailed procedures for the synthesis of the ligands and their corresponding Re complexes is described in the ESI.†

### Crystallographic data

**Fig. 2:**
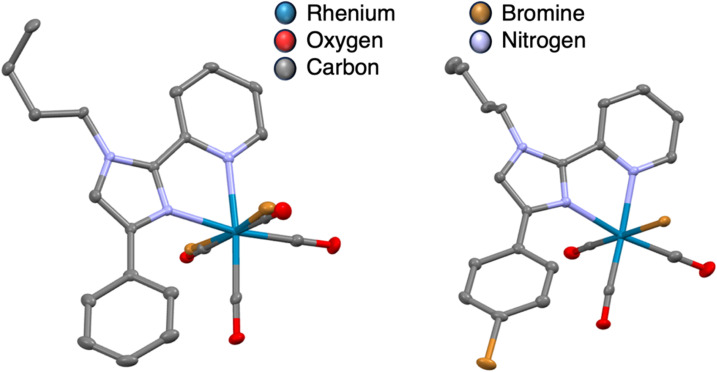
Crystal structures of RC2 (left) RC3 (right) omitting hydrogen atoms for clarity.

Suitable single crystals of RC2 and RC3 were obtained *via* vial-in-vial vapor diffusion using methylene chloride in the inner vial and diethyl ether in the outer vial after allowing the set-up to stand overnight (Fig. 2). Both crystals were bright yellow in color. Other instances of diimine pyridyl-imidazole rhenium crystals have been reported in the literature and a comparison of the selected bond lengths with a reference complex can be found below. The crystallographic data are summarized in Table 1. The Re1–N_imidazole_ and Re1–N_pyridine_ bond lengths are slightly shorter (0.005 Angstroms) in RC2 than RC3, likely owing to the electron withdrawing nature of the bromine substituent lengthening the Re–N bonds in RC3. The N_imidazole_–Re bond length in RC2 (2.181(2) Å) is longer than an analogous bond found in a benzimidazolylpyridine Re(i) complex (2.130(1) Å), indicating a weaker N–Re bond for the imidazole donor of RC2.^32,33^ Similarly, in a Re–pyridylimidazole complex with a 4-(*N*,*N*-dimethylamino)pyridine group instead of a bromine substituent, the N_imidazole_–Re bond length is shorter as well (2.118 Å). This indicates that in RC2/RC3, the bond lengths are longer than those observed for related Re(azolylpyridine) complexes.

**Table 1:** Crystallographic data of catalysts RC2 and RC3

	RC2	RC3
Empirical formula	C_21_H_19_BrN_3_O_3_Re	C_21_H_18_Br_2_N_3_O_3_Re
Formula weight	627.50	706.40
Crystal system	Orthorhombic	Monoclinic
Space group (no.)	*Pbca*	*P*2_1_/*c*
*a* (Å)	14.4135 (6)	11.7322 (3)
*b* (Å)	13.9657 (5)	11.2485 (2)
*c* (Å)	19.9434 (9)	17.8809 (4)
*α* (deg)	90	90
*β* (deg)	90	104.108
*γ* (deg)	90	90
*V* (Å^3^)	4014.5 (3)	2288.56
*Z*	8	4
*F*(000) (e)	2400	1336
*μ* (Mo-Kα) (mm^−1^)	8.073	
Reflections collected	43 216	46 047
Independent reflections	4593 (*R*_int_ = 0.0474)	5254 (*R*_int_ = 0.0310)
Observed reflections (*F*_o_ ≥ 2*σ*(*F*_o_))	43 216	46 047
Refined parameters	280	273
Goodness-of-fit on *F*^2^	1.163	1.057
*R*_a_, *R*_wb_ (*I* ≥ 2*σ*(*I*))	0.0195, 0.0434	0.0149, 0.0292
*R*_a_, *R*_wb_ (all data)	0.0212, 0.0440	0.0179, 0.0299

### UV-Vis analysis

**Fig. 3:**
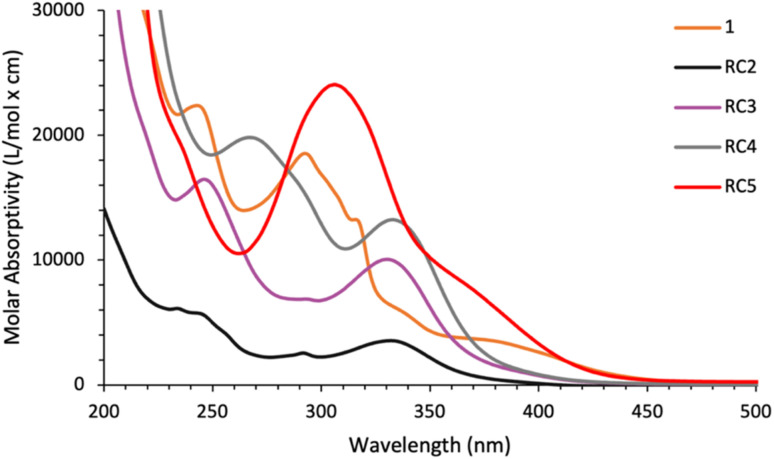
UV-Vis absorption spectra for catalysts 1–RC5 dissolved in MeCN.

To characterize the light absorption properties of each complex, UV-visible absorption spectra were collected of the complexes dissolved in acetonitrile (MeCN) and are shown in Fig. 3. The optical properties of the complexes are summarized in Table 2. The data from these experiments revealed that the absorption onsets (*λ*_onset_) of RC2–4 are blue-shifted compared to 1, in contrast to our hypothesis. We reason the blue shifts are due to the increased basicity of the imidazole donor in comparison to pyridine, in addition to the lower degree of conjugation in the imidazole ring. Complex RC5 shares a similar *λ*_onset_ to 1 at 425 nm. From the compounds, RC5 possesses the largest molar absorptivity coefficient while also possessing the most red-shifted MLCT absorption band in the visible light region. Interestingly, complexes RC2 and RC3 differ considerably in their molar absorptivity (Δ*ε* = 5853 M^−1^ cm^−1^) with the substitution of just one hydrogen for a bromine atom.

**Table 2:** Optical properties of catalysts 1 and RC2–5^a^

Catalyst	*λ*_max_ (nm)	*λ*_onset_ c (nm)	*ε* (M^−1^ cm^−1^)	*E*_MLCT-GS_ d (eV)
1	292, 384^b^	420	18 951	2.95
RC2	248, 337^b^	370	3718	3.35
RC3	246, 331^b^	375	9571	3.31
RC4	267, 336^b^	380	19 855	3.26
RC5	306, 371^b^	425	23 381	2.92

a Measured in acetonitrile.

b Indicates shoulder.

c Estimated from the intercept of the baseline and a tangent line on the UV-Vis spectrum on the low energy side of the absorption.

d Estimated from the onset of the low energy side of the absorption curve. The *E*_MLCT-GS_ was converted from nm to eV using the equation 1240/*E*_MLCT-GS_ nm = *E*_MLCT-GS_ eV.

### Electrochemical analysis

**Fig. 4:**
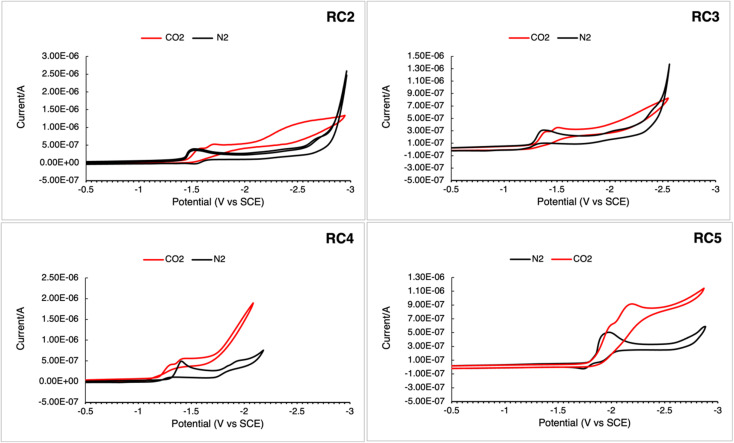
Cyclic voltammograms of catalysts RC2–RC5. Spectra were recorded in dry MeCN, 0.1 M Bu_4_NPF_6_ in MeCN using a Pt pseudo-reference, Pt counter, and glassy carbon working electrode using a scan rate of 0.1 V s^−1^. All spectra were referenced to ferrocene (Fc) as an internal standard using the Fc^+^/Fc = 0.4 V couple *vs.* SCE.^38^

The redox properties of RC2–5 were measured using cyclic voltammetry (CV) to examine whether the complexes could serve as CO_2_ reduction catalysts under a CO_2_ atmosphere and shown in Fig. 4 and summarized in Table 3. When CO_2_ was sparged into each solution of dissolved complex, there was an increase in current associated with the first reduction potential, indicative of catalytic CO_2_ reduction activity. The increase in current for complexes RC2 and RC3 was also accompanied by shifts in the reduction wave to higher energy, while the current for RC5 was accompanied by a shift in the reduction wave to a lower energy.

**Table 3:** Electrochemical properties of catalysts RC2–RC5^a^

Catalyst	*E*_(S+/S)_ b (V)	*E*_(S−/S)_ c (V)	*E*_(S*/S−)_ d (V)
1 (ref. 11)	1.26	−1.20	1.33
RC2	1.40	−1.35	2.00
RC3	1.45	−1.30	2.01
RC4	1.40	−1.35	1.91
RC5	0.85	−1.55	1.59
[Ru(bpy_3_)]^2+^ (ref. 34)	1.29	−1.33	0.31
*fac*-Ir(ppy)_3_ (ref. 34)	0.73	−2.19	0.34

a Measured in dry MeCN. All values are reported *vs.* SCE and were measured by CV in 0.1 M Bu_4_NPF_6_ in MeCN using a Pt pseudo-reference, Pt counter, and a glassy carbon working electrode.

b Estimated as the onset of the oxidation peak due to irreversibility of the wave.

c Estimated as the onset of the reduction peak due to irreversibility of the wave.

d Estimated *via* the sum of the reduction potential and *E*_MLCT-GS_ using the equation: *E*_(S*/S−)_ = *E*_(S−/S)_ + *E*_MLCT-GS_.

**Fig. 5:**
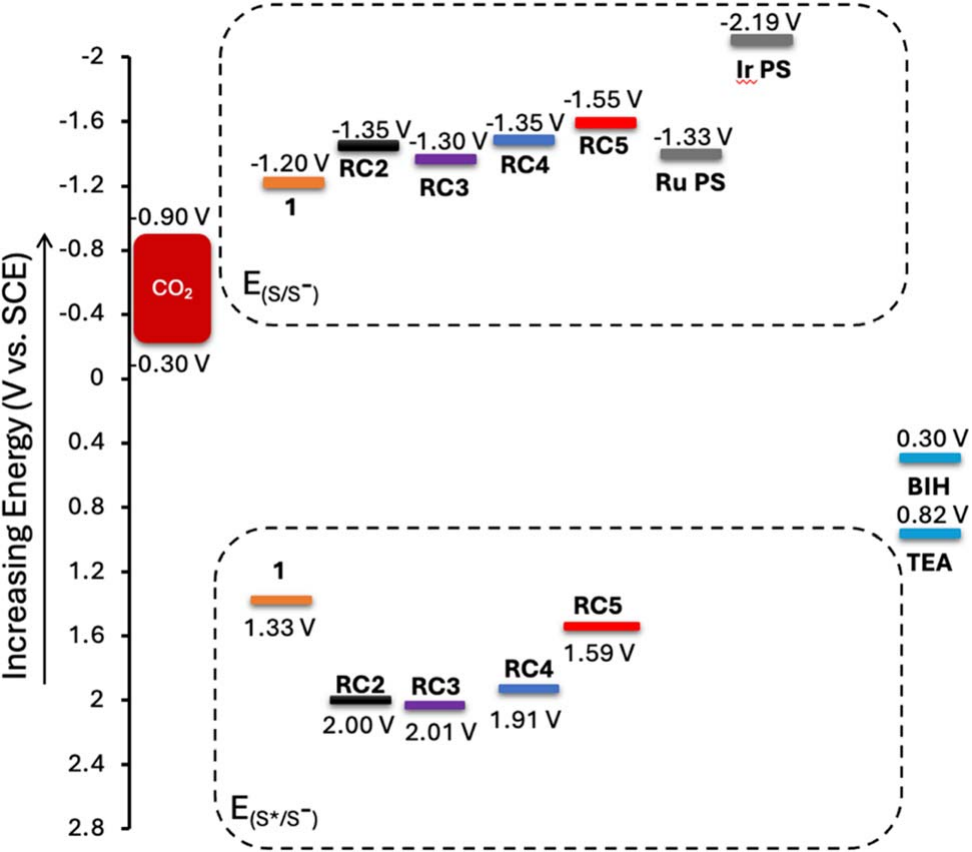
Energy level diagram illustrating of catalyst reduction potentials, calculated excited-state reduction potentials from the *E*_MLCT-GS_ values, approximated range for the standard 2e^−^/2H^+^ reduction potential of CO_2_ to CO (and H_2_O) in acetonitrile based on the minimum and maximum pH range in the photocatalytic trials, and the oxidation potentials of BIH and TEA.^35–37^

These measurements are also useful for determining whether the excited state reduction potentials of each complex are appropriately positioned for electron transfer from sacrificial electron donors like BIH or from photosensitizers (PS) such as Ir(ppy)_3_ or [Ru(bpy_3_)]^2+^. An energy level diagram showing relevant reduction potentials associated with the rhenium catalysts, the aforementioned photosensitizers, the standard reduction potential of CO_2_ to CO, and sacrificial electron donors BIH and TEA is given in Fig. 5. The reduction potentials of the dissolved complexes in nitrogen-sparged MeCN fall between −1.30 and −1.55 V *vs.* saturated calomel electrode (SCE), all of which are relatively higher in energy than the reduction potential of Re(bpy)(CO)_3_Br. The reduction potentials are also lower or very nearly lower in energy than both Ir(ppy)_3_ or [Ru(bpy_3_)]^2+^, which suggests that either could serve as a PS for the complexes. The excited state reduction potential of each complex falls between 1.59 and 2.01 V *vs.* SCE, which are all substantially lower than the oxidation potentials of both BIH and triethanolamine (TEOA) or triethylamine (TEA), which indicates that each can serve as an electron donor.

### Photocatalytic CO_2_ reduction

The Re complexes were applied to the photocatalytic reduction of CO_2_ using anhydrous MeCN as the reaction solvent and a natural white light LED light source (*λ* > 400 nm) adjusted to 1 Sun intensity. Following initial optimization experiments (Table S2†) with catalyst RC2 and RC5, [Ru(bpy)_3_]^2+^ was selected as a PS due to its superior performance for photocatalytic CO_2_ reduction compared to Ir(ppy)_3_. A sacrificial electron donor BIH was used with TEOA where TEOA plays a secondary role as a base in deprotonating the BIH radical cation generated in the initial electron transfer process to the excited PS to limit back electron transfer.^39^ TEOA was chosen as the amine to pair with BIH as it gave better activity compared to another commonly used base, TEA. TEOA has been shown to react with CO_2_ to form a zwitterionic alkylcarbonate adduct that serves to capture CO_2_ in solution and facilitate proton transfer events.^40^ Gaseous products such as carbon monoxide (CO), methane (CH_4_), and hydrogen (H_2_) were detected and quantified by gas chromatography (GC). Formic acid (HCO_2_H) as a solution-phase product of the photocatalytic reactions was quantified by ^1^H NMR spectroscopy using ferrocene as an internal standard. The detailed procedure for quantifying HCO_2_H and a representative ^1^H NMR spectrum for this measurement is given in the ESI (Fig. S7†).

**Fig. 6:**
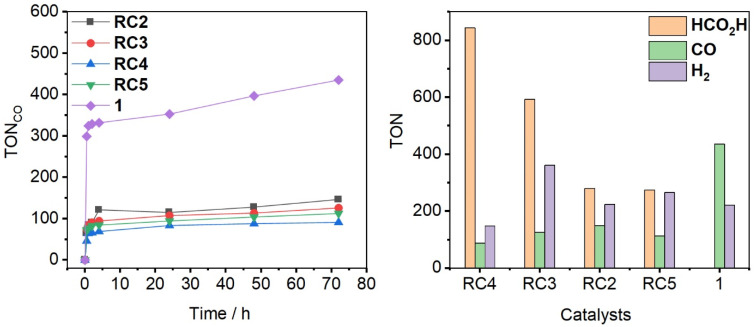
CO formation over 72 h period (left) and the production of HCO_2_H, CO, and H_2_ after 72 h (right) in photocatalytic CO_2_ reduction reaction with catalysts RC2, RC3, RC4, RC5, and 1. All experiments were performed with 1 μM catalyst, 50 mM BIH, 0.1 mM [Ru(bpy)_3_]^2+^, and 5% (v/v) TEOA in anhydrous MeCN solution. The data points given are the average of two sample runs.

The results of photocatalytic CO_2_ reduction experiments are summarized and shown in Table 4 and Fig. 6. In the presence of PS [Ru(bpy)_3_]^2+^ and electron donor BIH, Re-complexes RC2–5 were found to be active in light-driven CO_2_ reduction with observed products HCO_2_H, CO, and H_2_. At a catalyst concentration of 1 μM, CO_2_ is primarily reduced to HCO_2_H as the dominant product, accompanied by H_2_ as a secondary byproduct from proton reduction. A smaller amount of CO was also formed. Interestingly, catalyst RC4, having resonance π-A group, exhibited the best catalytic performance for CO_2_ reduction with a turnover number (TON) value of 844 for HCO_2_H production and the highest selectivity for CO_2_ reduction products of 86% (Table 4, entry 1 and Fig. 6 (right)). Catalysts RC2 (TON for HCO_2_H = 280) and RC5 (TON for HCO_2_H = 275) with π-bridge and π-D group, respectively, showed lower catalytic activity overall than RC4, but similar activities and selectivities to one another with RC2 having a modestly higher carbon selectivity percentage (Table 4 and Fig. 6). Complex RC3 with Br-substituted π-bridge gave the second highest TON value for HCO_2_H of 593, which exceeds RC5 and the structurally similar complex RC2, but also produced the largest TON value for H_2_ at 362. This photocatalytic study establishes a clear correlation between the electron withdrawing π-A (–PhCN) and electron donating π-D (–NPh_2_) substituents attached to the imidazole ring. Among the catalysts investigated, the best performance was observed from the Re complex RC4 which contains a π-A group, followed by less electron withdrawing Br-substituted π-bridge RC3. Electronically neutral catalyst RC2 and π-D group substituted RC5 showed the lowest TON value for HCO_2_H generation. Notably, no HCO_2_H was observed for benchmark catalyst 1. A recent publication described that [Re(bpy)_2_(CO)_2_]^+^ also selectively formed HCO_2_H with a TON value of 428 from CO_2_ reduction, and they proposed a catalytic cycle similar to that of ruthenium catalyst [Ru(bpy)_2_(CO)_2_]^2+^.^28^ Doyle and coworkers have reported a photocatalyst, Re(CO)_3_(1-(1,10)phenanthroline-5-(4-nitro-naphthalimide))Cl, which is selective for HCO_2_H generation with a TON value of 533.^41^ We propose that the catalysts reported here also utilize the same catalytic cycle as that reported by Richeson and coworkers, which features a Re–H intermediate that undergoes CO_2_ insertion to give a Re–formato intermediate.^28^ Each of the imidazole-pyridine based catalysts reported here generate a mixture of HCO_2_H, CO, and H_2_ with HCO_2_H being the dominant product overall; likewise, TON values for H_2_ are greater than those of CO. The highest TON values for HCO_2_H production were obtained with catalysts bearing electron withdrawing substituents (RC3 and RC4) on the imidazole donor with HCO_2_H : H_2_ ratios of 5.7 (RC4) and 1.6 (RC3). The catalysts with more electron donating substituents (RC2 and RC5) slightly favor HCO_2_H, but with lower TON values and HCO_2_H : H_2_ ratios closer to 1. A rhenium-hydride species^28^ is expected to be a common intermediate responsible for the production of HCO_2_H and H_2_ during catalysis, both of which are formed in higher quantities than CO. The competition between HCO_2_H and H_2_ suggests that the hydricity of the putative Re–H is near 44 kcal mol^−1^ (*i.e.* the hydricity of formate in acetonitrile)^42^ and sensitive to the ligand substitutions. To the best of our knowledge, Re complex RC4 sets a new benchmark with the highest TON value for HCO_2_H production of 844 reported for rhenium-based CO_2_ reduction catalysts.

**Table 4:** Performance comparison of the catalysts in the photocatalytic reduction of CO_2_^a^

Entry	Catalyst	HCO_2_H (TON^b^)	CO (TON^b^)	H_2_ (TON^b^)	CS^c^ (%)
1	RC4	844	88	148	86
2	RC3	593	126	362	67
3	RC2	280	150	224	66
4	RC5	275	113	266	59
5	1^d^	0	436	222	67

a Reactions were conducted in anhydrous MeCN with 1 μM catalyst/2 mL, 0.1 mM [Ru(bpy)_3_]^2+^, 50 mM BIH, and 5% (v/v) TEOA/MeCN.

b Turnover number (TON) values are reported at 72 h after catalysis was no longer occurring.

c Carbon-selective (CS) reduction percentage is calculated as CS% = [(CO TON + CH_4_ TON + HCO_2_H TON)/total observed product TON × 100].

d Previously published; no HCO_2_H formation.

Under the same conditions, complex 1 outperformed catalyst RC2–5 regarding CO production (Fig. 6 and Table 4, entry 5). Comparing catalysts RC2–5, catalyst RC2 with a π-bridge on the imidazole ring showed comparatively better catalytic activity with a TON value of 150 for CO generation. In contrast, RC4 exhibited a relatively low TON value of 88 for CO evolution, strongly favoring HCO_2_H production. Toward H_2_ production, the highest activity (TON_H_2__ = 362) was observed for complex RC3, followed by RC2 and RC5, with complex RC4 giving the lowest TON value for H_2_ (Table 4).

Catalytic performance of RC2–5 was also evaluated without using an external PS (Table S1†). Although the parent complex 1 is known to exhibit self-sensitized catalytic activity (TON_CO_ = 55), only one of the Re complexes (RC5) absorbed visible light. Each of the catalysts exhibited very poor self-sensitized photocatalytic activity. Therefore, we conclude that catalysts RC2–RC5 required a PS for significant catalysis to occur.

The influence of catalyst concentration on TON values was also investigated using complexes RC4 and RC2 (Table 5). The TON values decreased dramatically when the catalyst concentration was changed from 1 μm to 10 and 100 μM. This is a typical result, as lowering the catalyst concentration has been shown to result in higher TON values owing to the catalytic sites being isolated from one another, therefore reducing deleterious side reactions.^43,44^

**Table 5:** Results of photocatalytic CO_2_ reduction with RC2 and RC4 at different catalyst concentrations^a^

[Catalyst] (μM)	Catalyst	HCO_2_H (TON)	CO (TON)	H_2_ (TON)	CS^b^ (%)
1	RC4	844	88	148	86
	RC2	280	150	224	66
10	RC4	53	16	18	79
	RC2	20	69	29	75
100	RC4	17	8	3	89
	RC2	8	6	5	74

a Maintained a constant irradiation time of 72 h and the same reaction conditions provided in Table 1.

b CS% = [(CO TON + CH_4_ TON + HCO_2_H TON)/total observed product TON × 100].

The control experiments were performed using complex RC2 to determine the necessity of each component used in the photocatalytic CO_2_ reduction. The outcomes of these experiments are summarized in Table 6, where entry 1 is the result of our standard condition (all components present). Photocatalysis under N_2_ in the absence of CO_2_, or without the rhenium catalyst, or without irradiation (entries 5–7), resulted in negligible product formation, indicating the necessity of the substrate, the Re catalyst, and light, respectively. The impact of removing the PS (TON_HCO_2_H_ with PS = 280 and without PS = 0, entries 1 and 2) and removing the electron donor BIH (TON_HCO_2_H_ with BIH = 280 and without BIH = 10, entries 1 and 3) is significant. BIH is a better electron donor (more driving force for electron transfer) than TEOA, and thus, better catalytic activity is observed in the presence of BIH compared to using TEOA alone as the sacrificial electron donor. Interestingly, in the absence of TEOA, catalyst RC2 showed similar TON values for HCO_2_H compared to the experiment with TEOA (Table 6, entries 1 and 4). However, lower TON values for CO and higher values for H_2_ production are also observed without TEOA, indicating that the presence of TEOA strongly influences product selectivity.

**Table 6:** Summary of the photocatalytic control experiments with catalyst RC2^a^

Entry	Catalyst	PS^b^	e^−^ donor^c^	CO (TON)	H_2_ (TON)	HCO_2_H (TON)
1	2	Yes	BIH, TEOA	150	224	280
2	2	No	BIH, TEOA	2	9	0
3	2	Yes	TEOA	16	14	10
4	2	Yes	BIH	45	388	228
5^d^	2	Yes	BIH, TEOA	2	1	0
6	None	Yes	BIH, TEOA	1	1	0
7^e^	2	Yes	BIH, TEOA	0	0	0

a Irradiation time was constant at 72 h. The activity under standard conditions is provided in entry 1.

b PS = [Ru(bpy)_3_]^2+^.

c When both components are listed both are present.

d Under nitrogen atmosphere.

e No irradiation.

In Table 7, catalysts RC2–5 are compared to recent Re(i) catalysts applied to light-driven CO_2_ reduction in the presence of a sacrificial electron donor. Müller *et al.*^45^ recently synthesized three rhenium tricarbonyl catalysts that produce TON values for CO as high as 125 without the presence of an external PS and using TEOA as the sacrificial electron donor. These excellent TON_CO_ values were achieved by relatively simple modification to a 1,10-phenanthroline core by adding different heterocyclic substitutions at the 4 and 7 positions. Rotundo *et al.*^46^ also achieved similar TON_CO_ values of 120 with Re^I^(CO)_3_ catalysts using BIH instead of TEOA while studying the effects of substituted 2,2′-bipyridine based ligands containing various functional groups in the second-coordination sphere. Liang and coworkers^24^ reported a self-sensitized rhenium catalyst utilizing pendant dipyrromethene-BF_2_ chromophores linked to a 2,2′-bipyridine core to achieve TON_CO_ values as high as 1376 with BIH as the sacrificial electron donor. Although catalyst RC2 possesses similar TON_CO_ values to Müller's and Rotundo's catalysts, their compounds function as effective self-sensitized photocatalysts while RC2 does not. With respect to formic acid production, RC4 in particular performs remarkably well compared to most Re catalysts that are generally known for their high selectivity for CO_2_-to-CO conversion. However, there are two recent rhenium catalysts that preferentially produce HCO_2_H. Spear *et al.*^27^ recently synthesized a rhenium catalyst supported by a phenanthroline nitro-naphthalimide ligand which selectively produced HCO_2_H in the absence of an external PS with a TON value as high as 533. Hameed *et al.*^28^ also achieve high selectivity and a HCO_2_H TON value of 428 utilizing the triflate salt of a bis(bipyridine) dicarbonyl rhenium complex which becomes less selective for HCO_2_H over the course of the reaction. Catalyst RC4 in comparison yields greater TON values for HCO_2_H, but with lower selectivity due to the formation of CO and H_2_, products that are not generated in large quantities or at all with the other two catalysts.

**Table 7:** Summary of the catalytic performance of selected rhenium catalysts applied to light-driven CO_2_ reduction

Catalyst	PS	e^−^ donor	CO (TON)	H_2_ (TON)	HCO_2_H (TON)	CS^d^ (%)
RC4	[Ru(bpy)_3_]^2+^	BIH, TEOA	88	148	844	86
RC4	No	BIH, TEOA	5	1	0	85
*fac*-[Re(NN)^a^(CO)_3_Cl]^45^	No	TEOA	125	nd	nd	100
*fac*-[ReCl(L)^b^(CO)_3_]^46^	No	BIH	120	nd	nd	100
[Re(BDP)_2_^c^]^24^	[Ru(bpy)_3_]^2+^	BIH	1376	nd	nd	100
[Re(CO)_3_(1-(NN)^a^-5-(4-nitro-napthalimide))Cl]^27^	No	BIH	0.04	nd	533	100
*cis*-[Re(bpy)_2_(CO_2_)]^+^OTf^−^(1^+^OTf^−^)^28^	[Ru(bpy)_3_]^2+^	BIH, TEOA	nd	38	428	92

a NN = 1,10-phenanthroline having pyrrole.

b L = Ph-NH_2_ substituted 2,2′-bipyridine.

c BDP = 2,2′-bipyridine having dipyrromethene-BF_2_ chromophores.

d CS% = [(CO TON + HCO_2_H TON)/total observed product TON × 100].

## Conclusions

Carbon dioxide valorization to fuels or fuel precursors is an attractive strategy to reduce the overall use of non-renewable carbon fuel sources. Here, we synthesized four novel imidazolyl-pyridine Re complexes that were applied to the photocatalytic reduction of CO_2_ and compared to benchmark catalyst Re(bpy)(CO)_3_Br, 1. By replacing one of the pyridyl rings for a substituted imidazole group containing electron donating or accepting moieties, we were able to tune the electronic properties and light absorption of the complexes. Of note is that each of the imidazolyl complexes favored HCO_2_H production, which is unusual for Re(CO)_3_ based catalysts which overwhelming mediate the conversion of CO_2_-to-CO with high selectivity. Catalysts RC3 and RC4 strongly favored HCO_2_H generation, with RC4 being the most active and selective catalyst overall with an overall carbon selectivity (CS) of 86%. The CS percentage of RC3 is 67% due to a relatively large TON value for H_2_ evolution. Catalysts RC2 and RC5 also favor HCO_2_H as the dominant CO_2_ reduction product but make comparably higher amounts of CO and H_2_ and thus have lower carbon selectivity of 66 and 59%, respectively. In sharp contrast, benchmark catalyst 1 does not produce HCO_2_H under the same conditions. The production of HCO_2_H is rare for rhenium-based catalysts for CO_2_ reduction with only a few examples known in the literature. Catalyst RC4 gave a TON value for HCO_2_H as high as 844 at 1 μM catalyst concentration, further demonstrating the high stability of this catalyst under photochemical conditions.

## Data availability

The data supporting this article have been included as part of the ESI.† CCDC 2428908 and 2428909 contain the supplementary crystallographic data for this paper. These data can be obtained free of charge *via*http://www.ccdc.cam.ac.uk/data_request/cif, or by emailing data_request@ccdc.cam.ac.uk, or by contacting The Cambridge Crystallographic Data Centre, 12 Union Road, Cambridge CB2 1EZ, UK; fax: +44 1223 336033.

## Conflicts of interest

There are no conflicts to declare.

## Supplementary Material

RA-015-D5RA01561H-s001

RA-015-D5RA01561H-s002
