# Supplementary material for: Light-driven CO2 reduction with substituted imidazole-pyridine Re catalysts favoring formic acid production
Source: RSC Adv. 2025 Apr 22;15(16):12547–56. doi: 10.1039/d5ra01561h (PMC12012614; doi:10.1039/d5ra01561h)
Supplement: RA-015-D5RA01561H-s001 [file RA-015-D5RA01561H-s001.pdf]

Supplementary Information (SI) for RSC Advances.
This journal is © The Royal Society of Chemistry 2025

Light-Driven CO_2 Reduction with Substituted Imidazole-Pyridine Re Catalysts Favoring Formic Acid Production

(Supporting Information)

Ryan Chafin,[a,#] Majharul Islam Sujan,[b,#] Sean Parkin,[a] Jonah W. Jurss,[b] Aron J. Huckaba[a,*]
a: Department of Chemistry, University of Kentucky, Lexington, KY 40506, United States
b: Department of Chemistry and Biochemistry, University of Mississippi, University, Mississippi 38677, United States
#: co-first author

*Corresponding author. Email: aron.huckaba@uky.edu

Experimental Section

General Information: All commercially obtained reagents were used as received. Thin-layer chromatography (TLC) was conducted with Silicycle aluminum backed TLC silica gel F_{254} sheets and visualized with UV light. Flash column chromatography was performed using a Teledyne Isco CombiFlash Nextgen 300+ with ELSD Flash Chromatography System using SilicaFlash P60, 40-63 μm (230-400 mesh) silica. ^{1}H and ^{13}C NMR spectra were acquired at room temperature using Jeol-500 MHz and Bruker-400 MHz spectrometers and are reported in ppm using the residual protiated solvent as an internal standard ($CHCl_3$ at 7.26 ppm and DMSO at 2.50 ppm). NMR data are reported using the following notation: s = singlet, d = doublet, t = triplet, q = quartet, p = pentet, m = multiplet, b = broad, ap = apparent; coupling constant(s) in Hz; integration. UV-Vis spectra were measured with a Cary 5000 UV-Vis spectrometer. Cyclic voltammograms were measured with a CH Instruments electrochemical analyzer using ferrocene as an internal reference, platinum wire as both counter and reference electrodes, and a glassy carbon disk (3 mm dia.) as the working electrode. All cyclic voltammograms were performed in dry acetonitrile using 0.1 M tetrabutylammonium hexafluorophosphate as the supporting electrolyte with a scan rate of 0.1 V/s. All single−crystal data was collected on a Bruker D8 Venture κ−axis diffractometer with Mo Kα radiation (0.71073 Å) at 100 K. The crystal structures were solved by dual−space methods and refined by full−matrix least−squares using the SHELX programs.

Photocatalysis Procedure

A natural white light LED (Thor Laboratories, λ > 400 nm) was used as the light source for irradiation in photocatalytic experiments. The intensity of the light source was adjusted to 1 sun intensity (180 mW) with a Coherent FieldMate™ laser power meter with a Coherent PowerMax PM10 detector. Anhydrous acetonitrile was used to prepare stock solutions of the catalysts and photosensitizers. A stock solution of the catalysts and photosensitizers was carefully prepared using anhydrous acetonitrile before each set of experiments. Photocatalysis reactions were conducted in an overnight-dried Pyrex glass tube. In a typical photocatalysis run, the calculated amounts of the stock solutions of the catalyst, photosensitizer, BIH, and TEOA were added to the tube. Excess acetonitrile was added to the tube before sealing it with a rubber septum. The prepared solution was then bubbled vigorously with CO_2 or N_2 for at least 15 min or until reaching a final solution volume of 2 mL. The solution was then placed under the light source. One of the catalysts was used in control experiments where one key component was removed at a time. The headspace of the reaction tube was sampled periodically to detect and quantify the gaseous products. The solvated products were analyzed by ^{1}H NMR spectroscopy at the end of the reaction. All experiments were duplicated, and the reported turnover numbers are the average of the two runs.

Product Analysis in Photocatalytic Experiments

A custom PerkinElmer Clarus 680 Gas Chromatography (Agilent PorapakQ column, 6 ft, 1/8 in. OD) with a dual detector system (TCD and FID) was used to analyze gaseous products. The quantification of CO and CH_4 using an FID detector, while H_2 was measured at the TCD detector. All the standards of known concentration used in calibration were purchased from buycalgas.com. Formic acid (HCO_2H) was detected and quantified according to a previously reported procedure.[1] In this procedure, 0.4 mL of the photolysis solution was taken and degassed

with N_2 for 10 min. Fresh anhydrous acetonitrile was used to replace evaporated acetonitrile. The solution was then reacted in an ultrasonic bath for 10 to 15 min with 18 µL of DBU (1,8-diazabicyclo[5.4.0]undec-7ene). Next, 580 µL of ferrocene solution (1.19 mM solution in CD_3CN) was added to the mixture. After mixing thoroughly, a ^{1}H NMR spectrum (D1-delay = 10 seconds, numbers of scans = 200) was obtained. The ratio of the HCO_2H (~8.6 ppm) and ferrocene (~4.18 ppm) peaks were compared to a calibration curve prepared with a wide range of formic acid concentrations (0 mM, 1 mM, 5 mM, 10 mM, 20 mM). Following this method, the concentration of HCO_2H could be measured accurately where the linear fit on the calibration curve has an R^2 value of 0.9999 (**Figure S5**). All NMR spectra were analyzed with MestReNova software with baseline correction in the desired region prior to integration of the peaks. Turnover numbers for HCO_2H production were calculated using the calibration curve in **Figure S5**.

Synthesis of amino(pyridin-2-yl)methaniminium chloride (1):[2] To a flame-dried 150 mL round bottom flask charged with a stir bar, 2-cyanopyridine (2.00 g, 19.2 mmol, 1.0 equiv.), sodium methoxide (0.032 g, 0.600 mmol, 0.0313 equiv.), and dry, degassed methanol (96 mL) were added. The solution was refluxed overnight and monitored by TLC and once the starting material had been completely consumed, ammonium chloride (1.54 g, 28.8 mmol, 1.5 equiv.) was added and the solution was allowed to reflux overnight. An aliquot of the solution was subjected to TLC in DCM and revealed that the final product had been formed. The solution was concentrated under reduced pressure and ethanol (58 mL) was added to the solid and refluxed for an hour. After an hour, the solution was filtered hot and washed with a total of 25 mL of ethanol three times. The filtrate was collected and concentrated before the solid was triturated with 15 mL of cold chloroform. The mixture was filtered and the filter cake collected to yield a brown solid (2.22 g, 14.1 mmol, 73%). ^{1}H NMR (400 MHz, DMSO-d_6): δ 9.33 (b, 4H), 8.80 (d, J = 4.2 Hz, 1H), 8.49 (d, J = 7.97 Hz, 1H), 8.13 (td, J = 6.23, 1.58 Hz, 1H), 7.77 (dd, J = 5.12, 2.37 Hz, 1H).

Synthesis of *2-bromoacetophenone (2):*[3] To a flame-dried 250 mL round bottom flask charged with a stir bar, acetophenone (0.485 mL, 0.5 g, 4.16 mmol, 1.00 equiv.), *p*-toluene sulfonic acid monohydrate (0.749 g, 6.31 mmol, 1.50 equiv.), and acetonitrile (100 mL) *N*-bromosuccinimide (NBS) (0.749 g, 4.21 mmol, 1.01 equiv.) was dissolved in acetonitrile (100 mL) and added dropwise via an addition funnel over 50 minutes while the solution was coming to reflux. After refluxing for 2 hours, an aliquot of the solution was subjected to TLC in DCM and revealed that the product had formed almost completely. The solution was concentrated under reduced pressure and the residue was diluted with 50 mL of DCM. The organic layer was washed twice with water and concentrated. Then 10 mL of petroleum ether was added to triturate the solution and then it was filtered. The filter cake was washed with petroleum ether three times to ensure the unreacted starting material was removed. The crude product was subjected to flash chromatography using hexanes, a 1-minute gradient to 50% DCM in hexanes then a 10-minute gradient to 100% DCM to yield the desired product (0.567 g, 2.85 mmol, 69%). ^{1}H NMR (400 MHz, $CDCl_3$): δ 8.00 (d, J = 7.19 Hz, 2H), 7.62 (tt, J = 4.44, 1.81 Hz, 1H), 7.50 (t, J = 7.92 Hz, 2H), 4.46 (s, 2H).

Synthesis of *2-(4-phenyl-1H-imidazol-2-yl)pyridine (3):*[4] To a 50 mL round bottom flask charged with a stir bar, amino(pyridin-2-yl)methaniminium chloride (0.595 g, 3.77 mmol, 1.5 equiv.), THF and water (5 mL, 1.26 mL), and potassium bicarbonate (0.755 g, 7.54 mmol, 2 equiv.) were added and the solution was heated to reflux under nitrogen atmosphere. To an addition funnel,

2-bromoacetophenone (0.500 g, 2.51 mmol, 1.00 equiv.) and THF (25 mL) were added and the solution was added dropwise to the reaction mixture over a period of 40 minutes. The flask was covered in aluminum foil to prevent degradation of the alpha halo ketone. The reaction was monitored by TLC and the starting material was fully consumed after about 24 hours of refluxing. The flask was taken off from the heat and submerged in an ice bath to precipitate inorganic salts. The solution was filtered and subjected to flash chromatography with a gradient of 0 to 25% EtOAc in hexanes to yield the desired yellow solid (0.384 g, 1.74 mmol, 70.0%). ^{1}H NMR (400 MHz, DMSO-d_6): δ 12.92 (s, 1H), 8.62 – 8.55 (m, 1H), 8.10 (d, J = 7.9 Hz, 1H), 7.87 (dd, J = 9.5, 7.7 Hz, 3H), 7.72 (s, 1H), 7.34 (td, J = 6.9, 6.2, 2.0 Hz, 3H), 7.18 (t, J = 7.6 Hz, 1H).

Synthesis of 2-(1-butyl-4-phenyl-1H-imidazol-2-yl)pyridine (4):[5] To a flame-dried vial under nitrogen was charged a stir bar, 2-(4-phenyl-1H-imidazol-2-yl)pyridine (0.200 g, 0.904 mmol, 1.00 equiv.), dry, degassed, DMF (7.5 mL), 1-bromobutane (0.300 mL, 0.372 g, 2.71 mmol, 2.50 equiv.), and sodium hydride (60% in mineral oil) (0.108 g, 2.71 mmol, 2.50 equiv.). The solution was heated to 80 °C for 2 days. The reaction was monitored by TLC and after the starting material had been consumed, the solution was poured into 50 mL of water. The solution was extracted three times with DCM and the organic layer was washed with brine. The organic layer was collected and dried over anhydrous sodium sulfate. The solution was concentrated and subjected to flash chromatography using 25% EtOAc in hexanes to yield the desired product (0.150 g, 0.541 mmol, 60.0%). ^{1}H NMR (400 MHz, $CDCl_3$): δ 8.57 (d, J = 4.8, 1.6 Hz, 1H), 8.29 (d, J = 8.0, 1.2 Hz, 1H), 7.85 (dd, J = 8.2, 1.2 Hz, 2H), 7.75 (td, J = 7.8, 1.7 Hz, 1H), 7.38 (td, J = 7.7, 1.3 Hz, 2H), 7.29 (d, J = 1.1 Hz, 1H), 7.28 – 7.16 (m, 2H), 4.62 (t, J = 7.3 Hz, 2H), 1.81 (p, J = 8.3, 6.7 Hz, 2H), 1.36 (q, J = 7.4 Hz, 2H), 0.92 (t, J = 7.3, 1.1 Hz, 3H). ^{13}C NMR (126 MHz, $CDCl_3$): δ 150.93, 148.34, 144.71, 140.83, 136.62, 134.35, 128.67, 126.82, 124.95, 123.21, 122.45, 119.22, 77.45, 77.12, 76.94, 48.29, 33.50, 19.90, 13.80.

Synthesis of *2-bromo-1-(4-bromophenyl)ethan-1-one (5):*[6] To a flame dried 50 mL round bottom flask charged with a stir bar and submerged in an ice bath were added 4-bromoacetophenone (5.00 g, 25.12 mmol, 1.00 equiv.), dry, degassed, diethyl ether (15 mL), and aluminum trichloride (0.050 g, 0.015 mmol, 0.377 equiv.). An addition funnel was charged with dry, degassed, diethyl ether (10 mL) and bromine (1.29 mL, 4.01 g, 25.12 mmol, 1.00 equiv.), which were added dropwise to the reaction mixture over a period of 20 minutes. The reaction was monitored by TLC and after an hour most of the starting material had been consumed. The mixture was concentrated under reduced pressure with the assistance of a base trap to neutralize the HBr formed in situ. The residue was washed with 50 mL of 1:1 petroleum ether and water and the mixture was filtered. The filter cake was washed with small portions of water and petroleum ether until the orange color disappeared. The solid was left to dry on the filter funnel with suction and an off-white solid was collected once dried (4.93 g, 17.7 mmol, 71.0%). ^{1}H NMR (400 MHz, $CDCl_3$): δ 7.78 (d, 2H), 7.57 (d, 2H), 4.33 (s, 2H).

Synthesis of *2-(4-(4-bromophenyl)-1H-imidazol-2-yl)pyridine (6):*[4] To a 50 mL round bottom flask charged with a stir bar, amino(pyridin-2-yl)methaniminium chloride (0.426 g, 2.70 mmol, 1.5 equiv.), THF (3.6 mL), water (0.9 mL), and potassium bicarbonate (0.541 g, 5.40 mmol, 2 equiv.) were added and the solution was heated to reflux under nitrogen atmosphere. To an addition funnel were charged 2-bromo-1-(4-bromophenyl)ethan-1-one (0.500 g, 1.80 mmol, 1.00 equiv.) and THF (18 mL), which were added dropwise to the reaction mixture over a period of an

hour. The flask was covered in aluminum foil to prevent degradation of the alpha halo ketone. The reaction was monitored by TLC and the starting material was fully consumed around 24 hours of refluxing. The flask was taken off from the heat and submerged in an ice bath to precipitate inorganic salts. The solution was filtered and subjected to flash chromatography with a gradient of 0 to 25% EtOAc in hexanes to yield the desired yellow solid (0.384 g, 1.74 mmol, 70.0%). ^{1}H NMR (400 MHz, DMSO-d_6): δ 13.23 (s, 1H), 13.00 (s, 1H), 8.65 (d, J = 5.1 Hz, 1H), 8.62 (dt, J = 4.6, 1.4 Hz, 1H), 8.16 – 8.03 (m, 1H), 7.92 (td, J = 7.7, 1.8 Hz, 1H), 7.90 – 7.80 (m, 3H), 7.63 – 7.49 (m, 2H), 7.40 (ddd, J = 7.5, 4.8, 1.2 Hz, 1H).

Synthesis of *2-(4-(4-bromophenyl)-1-butyl-1H-imidazol-2-yl)pyridine (7):*[5] To a flame dried 50 mL round bottom flask charged with a stir bar and submerged in an ice bath were added 2-(4-(4-bromophenyl)-1H-imidazol-2-yl)pyridine (**6**) (0.400 g, 1.33 mmol, 1.00 equiv.), dry, degassed DMF (11 mL), sodium hydride (60% in mineral oil) (0.106 g, 2.66 mmol, 2.00 equiv.), and 1-bromobutane (0.287 mL, 0.364 g, 2.66 mmol, 2.00 equiv.). The solution was left to stir. The reaction was monitored by TLC and once all of the starting materials had disappeared the solution was poured into 50 mL of water. The solution was extracted three times with DCM and the organic layer was washed with brine. The organic layer was collected and dried over anhydrous sodium sulfate. The solution was concentrated and subjected to flash chromatography using 25% EtOAc in hexanes to yield the desired product. Yield: (0.364 g, 1.02 mmol, 77.0%). ^{1}H NMR (500 MHz, $CDCl_3$): δ 8.57 (d, J = 4.8, 1.8, 0.9 Hz, 1H), 8.27 (d, J = 8.0 Hz, 1H), 7.76 (td, J = 7.8, 1.9 Hz, 1H), 7.71 (d, 2H), 7.49 (d, 2H), 7.29 (s, 1H), 7.22 (ddd, J = 7.5, 4.9, 1.2 Hz, 1H), 4.62 (t, 2H), 1.81 (p, J = 8.8, 6.8 Hz, 2H), 1.44 – 1.29 (m, 2H), 0.92 (t, J = 7.4 Hz, 3H). ^{13}C NMR (101 MHz, $CDCl_3$): δ 149.58, 147.31, 143.74, 138.57, 135.53, 132.18, 130.56, 125.36, 122.06, 121.49, 119.27, 118.22, 76.34, 76.02, 75.70, 47.24, 32.33, 18.77, 12.65.

Synthesis of *4-(1-butyl-2-(pyridin-2-yl)-1H-imidazol-4-yl)-N,N-diphenylaniline (8):*[7] To a flame dried vial under nitrogen were charged a stir bar, 2-(4-(4-bromophenyl)-1*H*-imidazol-2-yl)pyridine (0.200 g, 0.561 mmol, 1.00 equiv.), diphenyl amine (0.190 g, 1.12 mmol, 2.00 equiv.), tri-tert-butylphosphonium tetrafluoroborate (0.003 g, 0.010 mmol, 0.018 equiv.), and dry degassed toluene (2.2 mL). The solution was degassed for 30 minutes. After 30 minutes, bis(dibenzylidineacetone)-palladium(0) (0.007 g, 0.008 mmol, 0.014 equiv.) and sodium tert-butoxide (0.108 g, 1.12 mmol, 2 equiv.) were added and the vial was heated to reflux. The reaction was monitored by NMR spectroscopy and once all the starting materials had been consumed, the mixture was concentrated and subjected to flash chromatography using a gradient of hexanes and DCM to yield an orange solid (0.188 g, 0.423 mmol, 76.0%). ^{1}H NMR (500 MHz, $CDCl_3$): δ 8.57 (d, 1H), 8.29 (s, 1H), 7.76 (d, 1H), 7.73 (d, J = 8.3 Hz, 2H), 7.26 – 7.18 (m, 5H), 7.10 (ddt, J = 9.7, 3.4, 2.5 Hz, 6H), 6.99 (tt, J = 7.3, 1.2 Hz, 2H), 4.62 (t, J = 7.4 Hz, 2H), 1.81 (p, J = 8.3, 6.4 Hz, 1H), 0.91 (t, J = 7.4 Hz, 3H). ^{13}C NMR (126 MHz, $CDCl_3$): δ 150.67, 148.45, 148.42, 147.88, 146.77, 144.87, 144.40, 139.70, 136.70, 136.68, 136.66, 133.26, 131.72, 129.28, 128.68, 126.53, 126.06, 125.01, 124.56, 124.17, 123.49, 123.25, 122.69, 122.65, 120.45, 119.35, 118.60, 77.41, 77.36, 77.15, 76.90, 48.38, 33.44, 19.88, 13.78.

Synthesis of *4'-acetyl-[1,1'-biphenyl]-3-carbonitrile (9):*[8] To a flame dried 250 mL round bottom flask charged with a stir bar and under nitrogen were added 4-bromoacetophenone (0.503 g, 2.51 mmol, 1.00 equiv.), 3-cyanophenylboronic acid (1.11 g, 7.53 mmol, 3.00 equiv.), dioxane and water (5:1, 193 mL, 7.7 mL), and sodium carbonate (2.00 g, 18.9 mmol, 7.5 equiv.). The

mixture was sparged with nitrogen for 20 minutes while stirring. After 20 minutes, tetrakis(triphenylphosphine)palladium(0), (0.143 g, 0.124 mmol, 0.05 equiv.) was added and the mixture was heated to reflux overnight. The reaction was monitored by TLC and when all of the starting materials had been consumed, the mixture was cooled to room temperature and filtered through a pad of celite that was washed with EtOAc several times. The filtrate was diluted with 100 mL of water and extracted three times with 50 mL of EtOAc each time. The combined organic layers were dried over sodium sulfate and concentrated under reduced pressure. The residue was subjected to flash chromatography using a gradient of hexanes to DCM to yield a tan solid (0.397 g, 1.79 mmol, 72.0%). ^{1}H NMR (400 MHz, $CDCl_3$): δ 8.00 (d, 2H), 7.83 (t, J = 1.8 Hz, 1H), 7.78 (dt, J = 7.8, 1.5 Hz, 1H), 7.66 – 7.56 (m, 3H), 7.52 (t, J = 7.8 Hz, 1H), 2.59 (s, 3H).

Synthesis of *4'-(2-bromoacetyl)-[1,1'-biphenyl]-3-carbonitrile (10):*[3] To a flame-dried 100 mL round bottom flask charged with a stir bar, 4'-acetyl-[1,1'-biphenyl]-3-carbonitrile (**9**) (0.400 g 1.81 mmol, 1.00 equiv.), *p*-toluene sulfonic acid monohydrate (0.517 g 2.72 mmol, 1.50 equiv.), and acetonitrile (45 mL) were added. *N*-Bromosuccinimide (NBS) (0.322 g, 1.81 mmol, 1.00 equiv.) was dissolved in acetonitrile (100 mL) and added dropwise via an addition funnel over 30 minutes while the solution was coming to reflux. After refluxing for 2 hours, an aliquot of the solution was subjected to TLC in DCM and revealed that the product had formed almost completely. The solution was concentrated under reduced pressure and the residue was diluted with 50 mL of DCM. The organic layer was washed twice with water and concentrated. The crude product was subjected to flash chromatography using a gradient of hexanes to DCM to yield the desired product (0.329 g, 1.10 mmol, 60.0%). ^{1}H NMR (400 MHz, $CDCl_3$): δ 8.04 (d, 2H), 7.84 (t, J = 1.8 Hz, 1H), 7.79 (dt, J = 7.8, 1.5 Hz, 1H), 7.68 – 7.60 (m, 3H), 7.54 (t, J = 7.8 Hz, 1H), 4.41 (s, 2H).

Synthesis of *4'-(2-(pyridin-2-yl)-1H-imidazol-4-yl)-[1,1'-biphenyl]-3-carbonitrile (11):*[4] To a 50 mL round bottom flask charged with a stir bar and under nitrogen, amino(pyridin-2-yl)methaniminium chloride (0.315 g, 2.00 mmol, 1.8 equiv.), THF (2.66 mL), water (0.67 mL), and potassium bicarbonate (0.400 g, 4.00 mmol, 2.00 equiv.) was added and the solution was heated to reflux. To an addition funnel were charged 4'-(2-bromoacetyl)-[1,1'-biphenyl]-3-carbonitrile (**10**) (0.339 g, 1.13 mmol, 1.00 equiv.) and THF (13.3 mL), which were added dropwise to the solution over a period of 40 minutes. The flask was covered in aluminum foil to prevent degradation of the alpha halo ketone. The reaction was monitored by TLC and the starting material was fully consumed after around 24 hours of refluxing. The flask was taken off from the heat and submerged in an ice bath to precipitate inorganic salts. The solution was filtered and subjected to flash chromatography with a gradient of 0 to 50% EtOAc in hexanes to yield the desired orange solid (0.274 g, 0.850 mmol, 75.0%). ^{1}H NMR (400 MHz, DMSO-d_6): δ 13.04 (s, 1H), 8.64 (s, 1H), 8.21 (s, 1H), 8.16 (d, J = 8.2 Hz, 1H), 8.08 (d, J = 8.1 Hz, 2H), 8.03 (d, J = 8.3 Hz, 1H), 7.95 (dt, J = 15.4, 7.8 Hz, 1H), 7.82 (q, J = 11.0, 7.7 Hz, 3H), 7.67 (t, J = 7.8 Hz, 1H), 7.59 (t, J = 6.2 Hz, 1H), 7.44 – 7.36 (m, 1H). ^{13}C NMR (101 MHz, DMSO-d_6): δ 166.51, 150.75, 149.51, 149.09, 148.93, 141.45, 138.12, 137.78, 136.18, 131.60, 131.26, 130.61, 130.35, 127.53, 126.93, 125.59, 123.71, 122.37, 120.21, 119.36, 112.57, 40.58, 40.37, 40.16, 39.95, 39.74, 39.53, 39.32.

Synthesis of *4'-(1-butyl-2-(pyridin-2-yl)-1H-imidazol-4-yl)-[1,1'-biphenyl]-3-carbonitrile (12):*[5] To a flame-dried vial under nitrogen and charged with a stir bar, 4'-(2-(pyridin-2-yl)-1H-

imidazol-4-yl)-[1,1'-biphenyl]-3-carbonitrile (0.150 g, 0.465 mmol, 1.00 equiv.), dry, degassed, DMF (4 mL), 1-bromobutane (0.100 mL, 0.127 g, 0.930 mmol, 2.00 equiv.), and sodium hydride (60% in mineral oil) (0.037 g, 0.930 mmol, 2.00 equiv.) were added. The solution was heated to 80 °C for 2 days. The reaction was monitored by TLC and once all of the starting materials had been consumed, the solution was poured into 50 mL of water. The mixture was extracted three times with DCM and the organic layer was washed with brine. The organic layer was collected and dried over anhydrous sodium sulfate. The solution was concentrated and subjected to flash chromatography using a gradient to 50% EtOAc in hexanes to yield the desired product (0.053 g, 0.140 mmol, 30.0%). ^{1}H NMR (500 MHz, $CDCl_3$): δ 8.59 (d, *J* = 4.9, 1.8, 0.9 Hz, 1H), 8.30 (d, *J* = 7.9, 1.3 Hz, 1H), 7.95 (d, 2H), 7.90 (t, *J* = 1.8 Hz, 1H), 7.85 (d, *J* = 7.7, 1.5 Hz, 1H), 7.78 (td, *J* = 7.7, 1.8 Hz, 1H), 7.63 – 7.56 (m, 3H), 7.53 (t, *J* = 7.8 Hz, 1H), 7.37 (s, 1H), 7.28 – 7.20 (m, 1H), 4.65 (t, *J* = 7.4 Hz, 2H), 1.83 (p, *J* = 8.8, 6.8 Hz, 2H), 1.43 – 1.32 (m, 2H), 0.93 (t, *J* = 7.4 Hz, 3H). ^{13}C NMR (126 MHz, $CDCl_3$): δ 150.80, 148.46, 145.01, 142.34, 140.01, 136.94, 136.69, 134.58, 131.30, 130.52, 129.66, 129.14, 128.33, 127.30, 125.56, 123.26, 122.63, 119.65, 119.07, 113.01, 77.37, 77.32, 77.12, 76.86, 48.41, 33.49, 19.91, 13.79.

Synthesis of 2-(1-butyl-4-phenyl-1H-imidazol-2-yl)pyridineTricarbonylbromorhenium(I) (13) (RC2):[9] To a flame dried vial under nitrogen and charged a stir bar, 2-(1-butyl-4-phenyl-1H-imidazol-2-yl)pyridine (0.069 g, 0.25 mmol, 1.00 equiv.), rhenium pentacarbonyl bromide (0.102 g, 0.25 mmol, 1.00 equiv.), and dry, degassed toluene (6.2 mL) were added and the flask was covered in aluminum foil and heated to reflux. The reaction was monitored by TLC and after 24 hours the starting material had been completely consumed. The solution was cooled to -7 °C for an hour and then filtered and washed with minimal amounts of cold toluene. The filter cake was dissolved in DCM and concentrated under reduced pressure to afford a bright yellow powder (0.085 g, 0.135 mmol, 55.0%). ^{1}H NMR (500 MHz, $CDCl_3$): δ 9.14 (d, *J* = 5.5 Hz, 1H), 8.02 (t, 1H), 7.83 (d, *J* = 8.2 Hz, 1H), 7.66 (dd, *J* = 7.1, 2.3 Hz, 2H), 7.51 – 7.47 (m, 3H), 7.42 (t, *J* = 6.6 Hz, 1H), 7.12 (s, 1H), 4.47 – 4.32 (m, 2H), 2.00 (p, *J* = 7.5 Hz, 2H), 1.58 – 1.53 (m, 2H), 1.05 (t, *J* = 7.4 Hz, 3H). ^{13}C NMR (126 MHz, $CDCl_3$): δ 154.87, 148.13, 146.51, 144.09, 138.77, 131.24, 130.02, 129.47, 128.72, 125.36, 122.45, 121.42, 77.36, 77.31, 77.11, 76.85, 49.56, 31.96, 20.00, 13.65. UV-Vis (MeCN): $\boldsymbol{\varepsilon} = 3718\ M^{-1}cm^{-1}$, λ_{max} = 248 nm, λ_{onset} = 370 nm. Cyclic voltammetry (0.1 M Bu_4NPF_6 in MeCN, sweep width 2.0-(2.6), 0.1 Vs^{-1} scan rate) vs SCE: $E(S/S^-)$ = -1.47 V; $E(S^+/S)$ = 1.35 V; E_g^{opt} = 3.35 eV.

Synthesis of *2-(4-(4-bromophenyl)-1-butyl-1H-imidazol-2-yl)pyridine Tricarbonylbromorhenium(I) (14) (RC3):*[9] To a flame dried vial under nitrogen and charged with a stir bar, 2-(1-butyl-4-phenyl-1H-imidazol-2-yl)pyridine (0.044 g, 0.124 mmol, 1.00 equiv.), rhenium pentacarbonyl bromide (0.050 g, 0.124 mmol, 1.00 equiv.), and dry, degassed toluene (6.2 mL) were added and the flask was covered in aluminum foil and heated to reflux. The reaction was monitored by TLC and after 24 hours the starting material had been completely consumed. The solution was cooled to -7 °C for an hour and then filtered and washed with minimal amounts of cold toluene. The filter cake was dissolved in DCM and concentrated under reduced pressure to afford a bright yellow powder (0.050 g, 0.071 mmol, 57.0%). ^{1}H NMR (500 MHz, $CDCl_3$): δ 9.14 (d, *J* = 5.6, 1.6, 0.9 Hz, 1H), 8.04 (td, *J* = 7.9, 1.6 Hz, 1H), 7.83 (dt, *J* = 8.3, 1.1 Hz, 1H), 7.66 – 7.60 (m, 2H), 7.58 – 7.51 (m, 2H), 7.44 (ddd, *J* = 7.7, 5.5, 1.2 Hz, 1H), 4.47 – 4.32 (m, 2H), 2.05 – 1.95 (m, 2H), 1.57 – 1.52 (m, 2H), 1.05 (t, *J* = 7.4 Hz, 3H). ^{13}C NMR (126 MHz, $CDCl_3$): δ 154.88, 147.93, 146.79, 142.90, 138.87, 131.96, 131.56, 130.08, 125.56, 123.99, 122.56, 121.53,

77.36, 77.31, 77.11, 76.85, 49.64, 31.94, 20.00, 13.65. UV-Vis (MeCN): $\boldsymbol{\varepsilon}$ = 9571 $M^{-1}cm^{-1}$, λ_{max} = 246 nm, λ_{onset} =375 nm. Cyclic voltammetry (0.1 M Bu_4NPF_6 in MeCN, sweep width 2.0-(2.6), 0.1 V s^{-1} scan rate) vs SCE: E(S/S^-) = -1.30 V; E(S^+/S) = 1.35 V; E_g^{opt} = 3.31 eV.

Synthesis of 4-(1-butyl-2-(pyridin-2-yl)-1H-imidazol-4-yl)-N,N-diphenylaniline Tricarbonylbromorhenium(I) (15) (RC5):[9] To a flame dried vial under nitrogen and charged with a stir bar, 4-(1-butyl-2-(pyridin-2-yl)-1*H*-imidazol-4-yl)-*N,N*-diphenylaniline (**8**) (0.071 g, 0.159 mmol, 1.00 equiv.), rhenium pentacarbonyl bromide (0.065 g, 0.159mmol, 1.00 equiv.), and dry, degassed toluene (6.0 mL) and the flask was covered in aluminum foil and heated to reflux. The reaction was monitored by TLC and after 24 hours the starting material had been completely consumed. The solution was cooled to -7 °C for an hour and then filtered and washed with minimal amounts of cold toluene. The filter cake was dissolved in DCM and concentrated under reduced pressure to afford a bright yellow powder (0.029 g, 0.036 mmol, 23.0%). ^{1}H NMR (400 MHz, $CDCl_3$): δ 9.03 (d, *J* = 5.4 Hz, 1H), 7.93 (td, *J* = 8.0, 1.6 Hz, 1H), 7.76 (d, *J* = 8.2 Hz, 1H), 7.43 – 7.37 (m, 2H), 7.32 (t, *J* = 6.6 Hz, 1H), 7.18 (dd, *J* = 7.7, 4.4 Hz, 4H), 7.13 – 7.03 (m, 6H), 7.03 (s, 1H), 6.97 (t, *J* = 7.3 Hz, 2H), 4.29 (qt, *J* = 14.6, 7.5 Hz, 2H), 1.90 (p, *J* = 7.5 Hz, 2H), 1.45 (dd, *J* = 15.4, 7.9 Hz, 3H), 0.96 (t, *J* = 7.3 Hz, 3H). ^{13}C NMR (126 MHz, $CDCl_3$): δ 196.99, 194.81, 189.30, 154.78, 149.10, 148.18, 147.57, 146.28, 143.83, 138.89, 130.90, 129.44, 129.16, 129.15, 128.34, 125.41, 125.25, 124.88, 124.84, 123.36, 123.13, 122.25, 121.52, 77.39, 77.34, 77.14, 77.12, 76.89, 49.51, 31.96, 19.98, 13.68. UV-Vis (MeCN): $\boldsymbol{\varepsilon}$ = 23381 $M^{-1}cm^{-1}$, λ_{max} = 306 nm, λ_{onset} = 425 nm. Cyclic voltammetry (0.1 M Bu_4NPF_6 in MeCN, sweep width 2.0-(2.6), 0.1 Vs^{-1} scan rate) vs SCE: E(S/S^-) = -1.55 V; E(S^+/S) = 0.85 V; E_g^{opt} = 2.92 eV.

Synthesis of 4'-(1-butyl-2-(pyridin-2-yl)-1H-imidazol-4-yl)-[1,1'-biphenyl]-3-carbonitrile Tricarbonylbromorhenium(I) (16) (RC4):[9] To a flame dried vial under nitrogen and charged with a stir bar, *4'-(1-butyl-2-(pyridin-2-yl)-1H-imidazol-4-yl)-[1,1'-biphenyl]-3-carbonitrile* (12) (0.057 g, 0.14 mmol, 1.00 equiv.), rhenium pentacarbonyl bromide (0.057 g, 0.14 mmol, 1.00 equiv.), and dry, degassed toluene (7.0 mL) and the flask was covered in aluminum foil and heated to reflux. The reaction was monitored by TLC and after 24 hours the starting material had been completely consumed. The solution was cooled to -7 °C for an hour and then filtered and washed with minimal amounts of cold toluene. The filter cake was dissolved in DCM and concentrated under reduced pressure to afford a bright yellow powder (0.061 g, 0.084 mmol, 60.0%). ^{1}H NMR (500 MHz, $CDCl_3$): δ 9.13 (dd, *J* = 5.6, 1.5 Hz, 1H), 8.04 (td, *J* = 8.0, 1.6 Hz, 1H), 7.89 (d, *J* = 1.8 Hz, 1H), 7.88 – 7.82 (m, 2H), 7.78 (d, *J* = 8.1 Hz, 2H), 7.68 (d, *J* = 8.1 Hz, 2H), 7.66 – 7.61 (m, 1H), 7.56 (t, *J* = 7.8 Hz, 1H), 7.47 – 7.41 (m, 1H), 7.19 (s, 1H), 4.49 – 4.33 (m, 2H), 2.00 (p, 2H), 1.59 – 1.50 (m, 2H), 1.04 (t, *J* = 7.4 Hz, 3H), 0.06 (s, 2H). ^{13}C NMR (126 MHz, $CDCl_3$): δ 154.88, 148.00, 146.81, 143.28, 141.90, 139.88, 138.90, 131.76, 131.32, 131.13, 130.82, 130.73, 129.84, 127.42, 125.53, 122.63, 121.57, 118.90, 113.11, 77.37, 77.32, 77.11, 76.86, 49.65, 31.96, 20.00, 13.65. UV-Vis (MeCN): $\boldsymbol{\varepsilon}$ = 19855 $M^{-1}cm^{-1}$, λ_{max} = 267 nm, λ_{onset} = 380 nm. Cyclic voltammetry (0.1 M Bu_4NPF_6 in MeCN, sweep width 2.0-(2.6), 0.1 Vs^{-1} scan rate) vs SCE: E(S/S^-) = -1.35 V; E(S^+/S) = 1.40 V; E_g^{opt} = 3.26 eV.

Synthesis of *2,2'-bipyridine Tricarbonylbromorhenium(I) (17) (Cat. 1):*[9] To a flame dried 100 mL round bottom flask under nitrogen and charged with a stir bar, 2,2'-bypyridine (0.044 g, 0.276 mmol, 1.00 equiv.), rhenium pentacarbonyl bromide (0.112 g, 0.276 mmol, 1.00 equiv.), and dry, degassed toluene (14 mL) and the flask was covered in aluminum foil and heated to reflux.

Once a yellow precipitate was observed (around 24 hours) the mixture was cooled to room temperature and filtered. The filter cake was dried and afforded a yellow solid (0.075 g, 0.148 mmol, 54.0%). ^{1}H NMR (400 MHz, $CDCl_3$): δ 9.03 (d, *J* = 5.4, 1.2 Hz, 5H), 8.14 (d, *J* = 8.1 Hz, 5H), 8.01 (td, *J* = 7.8, 1.5 Hz, 5H), 7.48 (t, *J* = 7.3, 5.6, 1.2 Hz, 5H).

UV-Vis Absorption Data for Each Catalyst

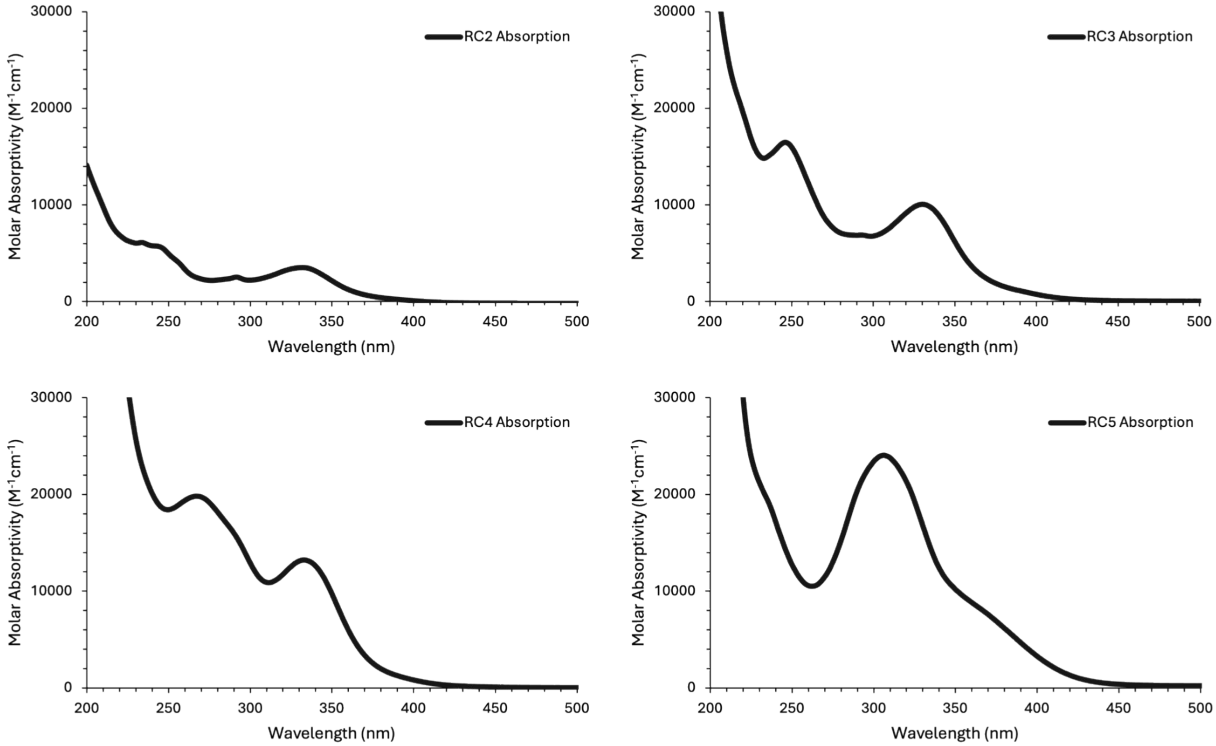

Figure S1. UV-Vis absorption spectra for complexes **RC2-RC5** in MeCN.

Cyclic Voltammetry Data

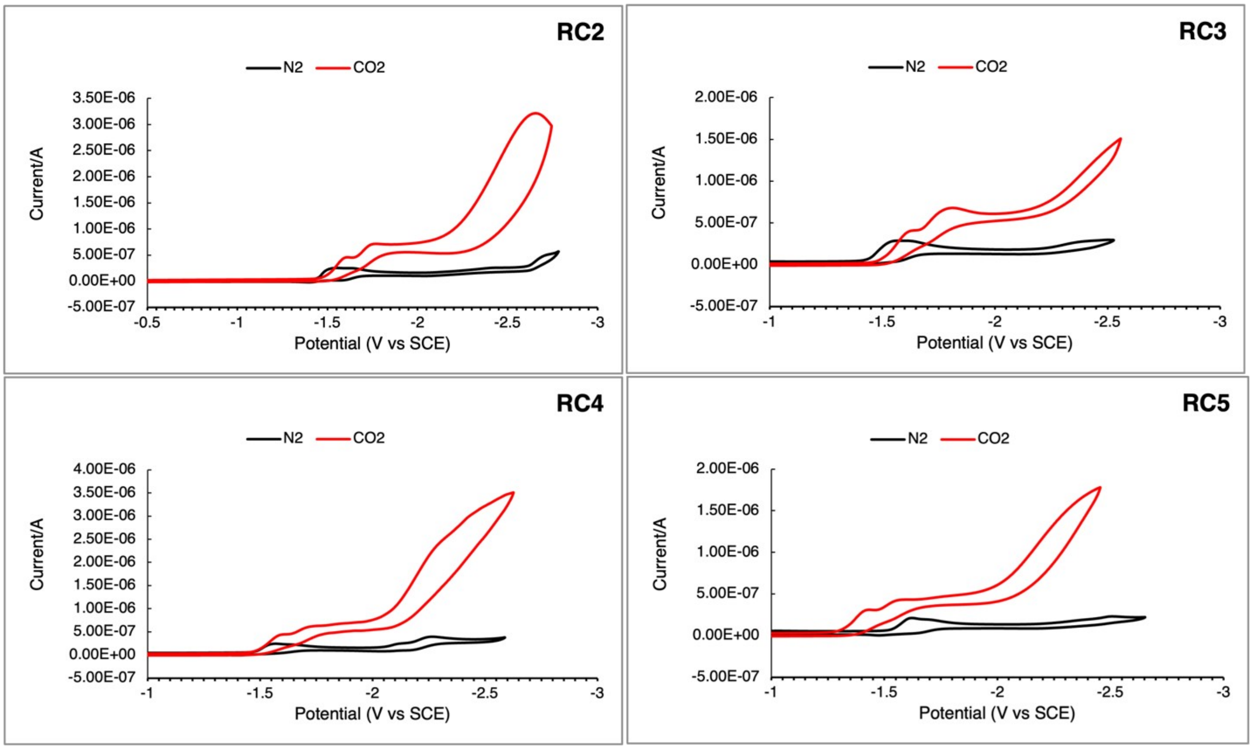

Figure S2. Cyclic voltammograms (CVs) of catalysts **RC2**-**RC5**. CVs were recorded in dry MeCN, 0.1 M NBu_4PF_6 in MeCN using a Pt pseudo-reference, Pt counter, and glassy carbon working electrode using a scan rate of 0.1 V/s. All CVs were referenced to Ferrocene (Fc) as an internal standard using the Fc/Fc^+ = 0.4 V couple vs. saturated calomel electrode (SCE). When changing atmospheres, the solutions were bubbled vigorously for at least 15 minutes each.

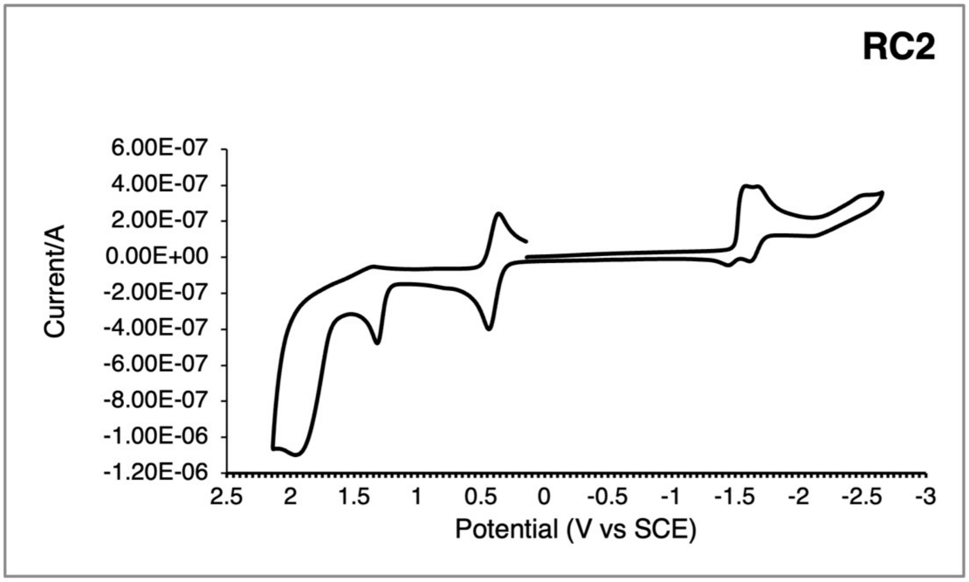

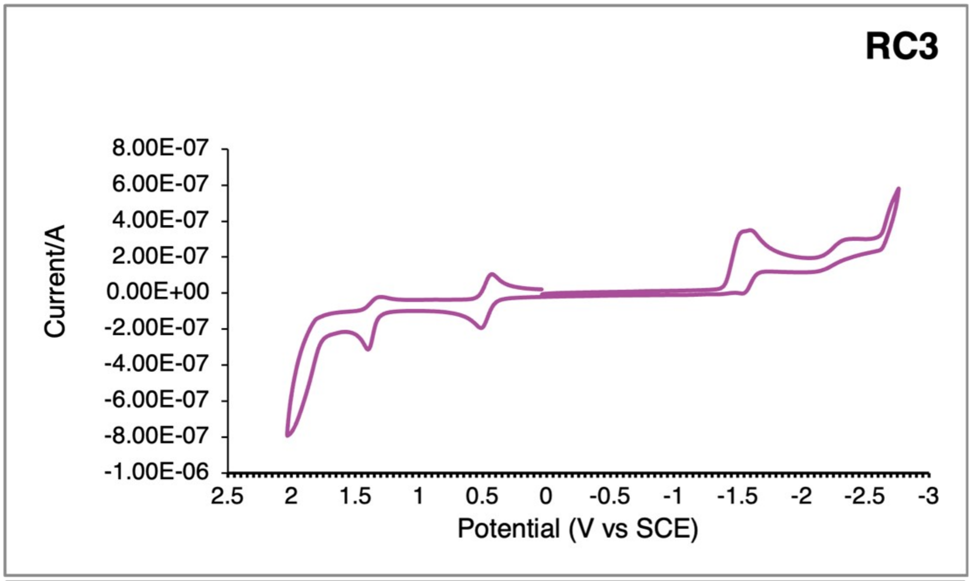
RC3
8.00E-07
6.00E-07
4.00E-07
2.00E-07
0.00E+00
-2.00E-07
-4.00E-07
-6.00E-07
-8.00E-07
-1.00E-06
Current/A
2.5
2
1.5
1
0.5
0
-0.5
-1
-1.5
-2
-2.5
-3
Potential (V vs SCE)

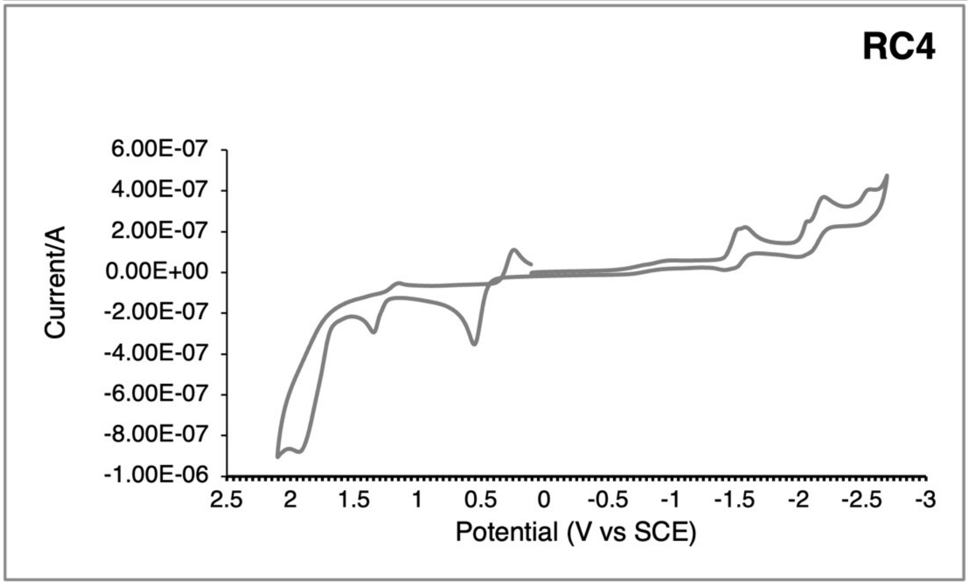
RC4
6.00E-07
4.00E-07
2.00E-07
0.00E+00
-2.00E-07
-4.00E-07
-6.00E-07
-8.00E-07
-1.00E-06
Current/A
2.5
2
1.5
1
0.5
0
-0.5
-1
-1.5
-2
-2.5
-3
Potential (V vs SCE)

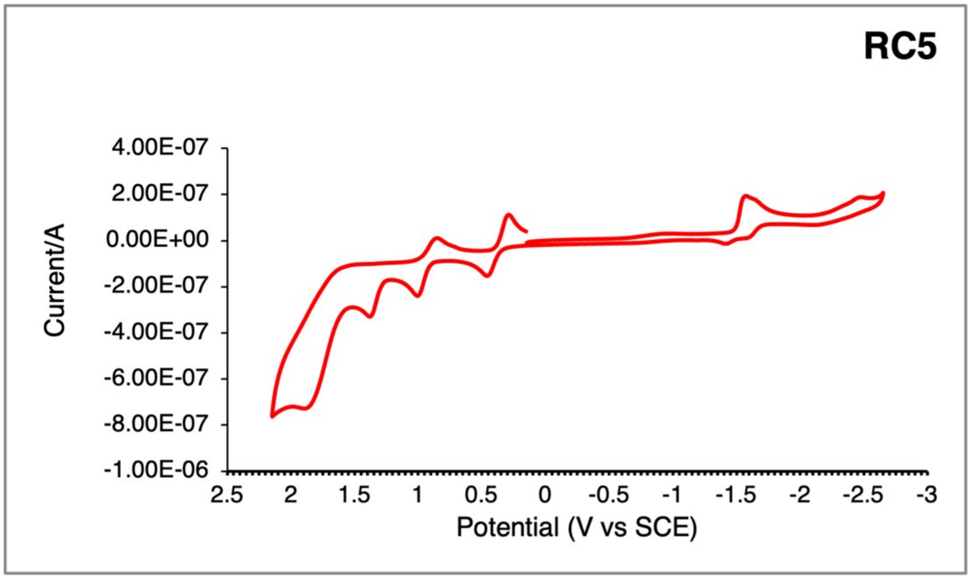

Figure S3. Cyclic voltammograms (CVs) of catalysts **RC2**-**RC5**. CVs were recorded in dry MeCN, 0.1 M NBu_4PF_6 in MeCN using a Pt pseudo-reference, Pt counter, and glassy carbon working electrode using a scan rate of 0.1 V/s. All CVs were referenced to Ferrocene (Fc) as an internal standard using the $Fc/Fc^+ = 0.4$ V couple vs. saturated calomel electrode (SCE).

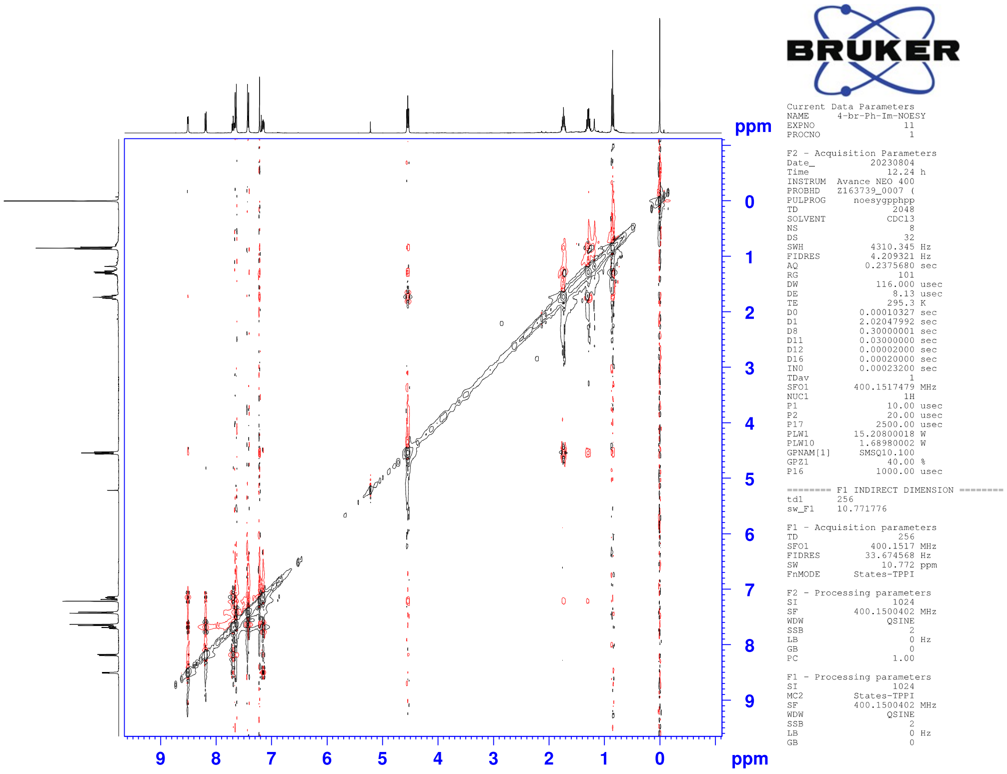

Figure S4. NOESY spectrum of compound **7** in $CDCl_3$.

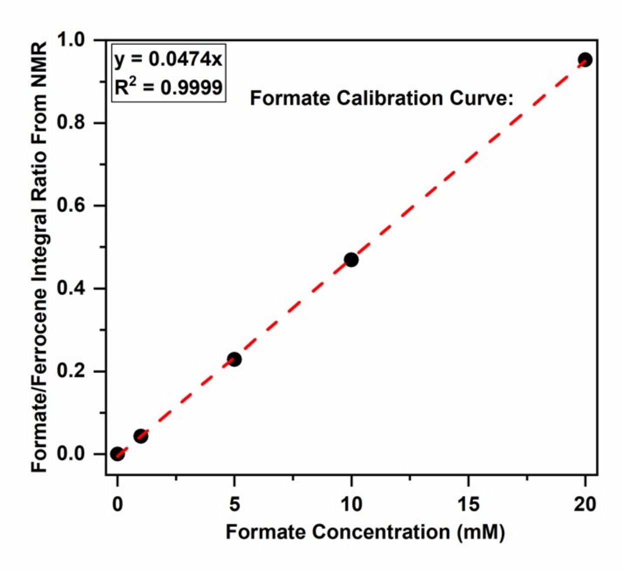

Figure S5: Calibration curve of the format quantification in d_3-MeCN with ferrocene as an internal standard for DBU.

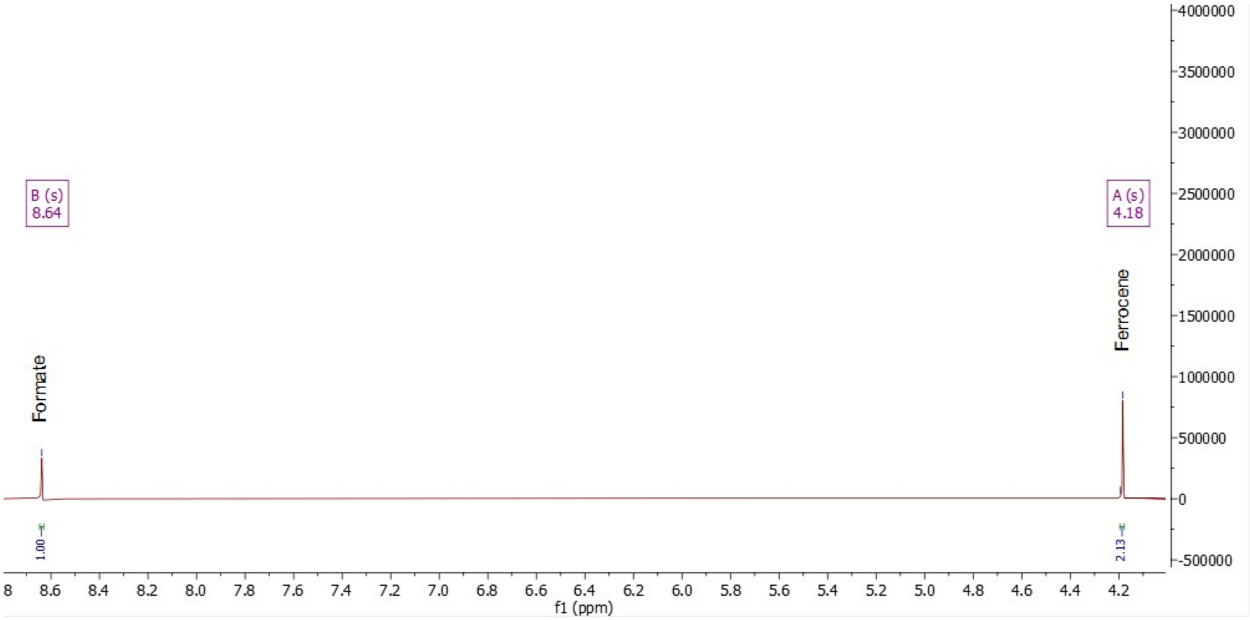

Figure S6: ^1H NMR spectrum that was used to create a calibration curve for HCO_2H in the presence of ferrocene as the internal standard and DBU as the base.

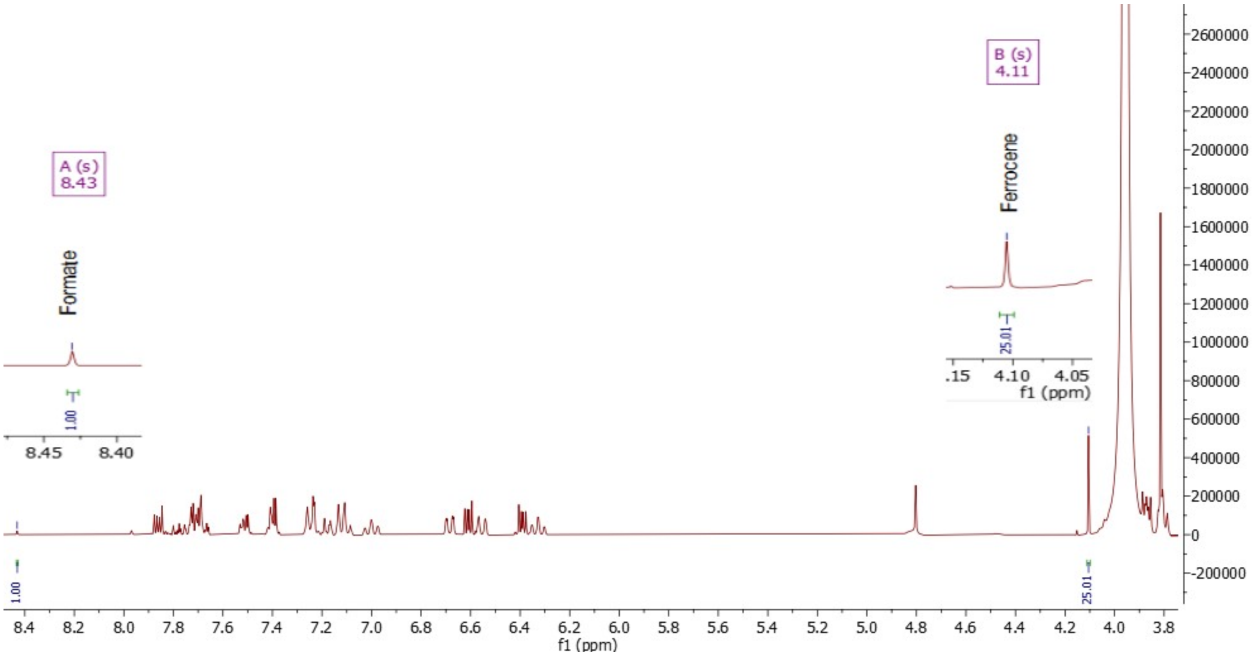

Figure S7: ^{1}H NMR spectrum of catalyst **RC4** (1 μM catalyst concentration) as a representative for HCO_2H detection and quantification in the presence of ferrocene as the internal standard and DBU as the base.

Table S1: Photocatalytic Performance of the Complexes without PS[a]

Catalyst	[Catalyst]	HCO_2H (TON)	CO (TON)	H_2 (TON)
**RC4**	0.1 mM	0	5	1
**RC3**	0.1 mM	0	1	0
**RC5**	0.1 mM	0	1	1
**RC5**	1 μM	0	2	10
**RC2**	0.1 mM	0	2	0
**RC2**	1 μM	0	2	9
**1**	0.1 mM	0	55	0

[a]Self-sensitized experiments were carried out in MeCN, BIH, and 5% TEOA. Reaction time was constant at 72 h.

Table S2: Optimization of the Best Condition for the Photocatalytic CO_2 Reduction[a]

Catalyst	[Catalyst] mM	PS	e^- donor[c]	CO (TON)	H_2 (TON)	HCO_2H (TON)
**RC2**	0.1	$[Ir(ppy)_3]^{2+}$	BIH, 5% TEA	2	3	0
**RC2**	0.1	$[Ir(ppy)_3]^{2+}$	BIH, 5% TEOA	2	5	0
**RC5**	0.1	$[Ir(ppy)_3]^{2+}$	BIH, 5% TEOA	1	5	0
**RC2**	0.1	$[Ru(bpy)_3]^{2+}$	BIH, 5% TEA	4	2	1
**RC2**	0.1	$[Ru(bpy)_3]^{2+}$	BIH, 5% TEOA	8	5	8
**RC2**	0.001	$[Ru(bpy)_3]^{2+}$	BIH, 5% TEOA	150	224	280

[a]Experiments were carried out in MeCN. Reaction time was constant at 72 h.

SI References

(1) Ha, E. G.; Chang, J. A.; Byun, S. M.; Pac, C.; Jang, D. M.; Park, J.; Kang, S. O. High-turnover visible-light photoreduction of CO2 by a Re(I) complex stabilized on dye-sensitized TiO2. Chem Commun (Camb) 2014, 50 (34), 4462-4464. DOI: 10.1039/c3cc49744e. https://doi.org/10.1039/C3CC49744E

(2) Kume, S.; Nomoto, K.; Kusamoto, T.; Nishihara, H. Intramolecular Electron Arrangement with a Rotative Trigger. Journal of the American Chemical Society 2009, 131 (40), 14198-14199. https://doi.org/10.1021/ja906684g.

(3) Lee, J. C.; Bae, Y. H.; Chang, S.-K. Efficient alpha-halogenation of carbonyl compounds by N-bromosuccinimide and N-chlorosuccinimde. Bulletin of the Korean Chemical Society 2003, 24 (4), 407-408. https://doi.org/10.1021/jo00911a026.

(4) PREPARATION OF 2,4-DISUBSTITUTED IMIDAZOLES: 4-(4-METHOXYPHENYL)-2-PHENYL-1H-IMIDAZOLE. *Org. Synth.* (81), 105. https://doi.org/10.1002/0471264229.os081.11

(5) Xu, T. H.; Lu, R.; Qiu, X. P.; Liu, X. L.; Xue, P. C.; Tan, C. H.; Bao, C. Y.; Zhao, Y. Y. Synthesis and Characterization of Carbazole-Based Dendrimers with Porphyrin Cores. European Journal of Organic Chemistry 2006, 2006 (17), 4014-4020. DOI: https://doi.org/10.1002/ejoc.200600356.

(6) Phenacyl Bromide. *Organic Syntheses* **1939,** *19*, 24. https://doi.org/10.15227/orgsyn.019.0024

(7) Hartwig, J. F.; Kawatsura, M.; Hauck, S. I.; Shaughnessy, K. H.; Alcazar-Roman, L. M. Room-Temperature Palladium-Catalyzed Amination of Aryl Bromides and Chlorides and Extended Scope of Aromatic C−N Bond Formation with a Commercial Ligand. The Journal of Organic Chemistry 1999, 64 (15), 5575-5580. https://doi.org/10.1021/jo990408i.

(8) Angello, N. H.; Rathore, V.; Beker, W.; Wołos, A.; Jira, E. R.; Roszak, R.; Wu, T. C.; Schroeder, C. M.; Aspuru-Guzik, A.; Grzybowski, B. A.; Burke, M. D., Closed-loop optimization of general reaction conditions for heteroaryl Suzuki-Miyaura coupling. *Science* **2022,** *378* (6618), 399-405. https://doi.org/10.1126/science.adc8743

(9) Thorp-Greenwood, F. L.; Platts, J. A.; Coogan, M. P. Experimental and theoretical characterisation of phosphorescence from rhenium polypyridyl tricarbonyl complexes. Polyhedron 2014, 67, 505-512. DOI: https://doi.org/10.1016/j.poly.2013.09.033.

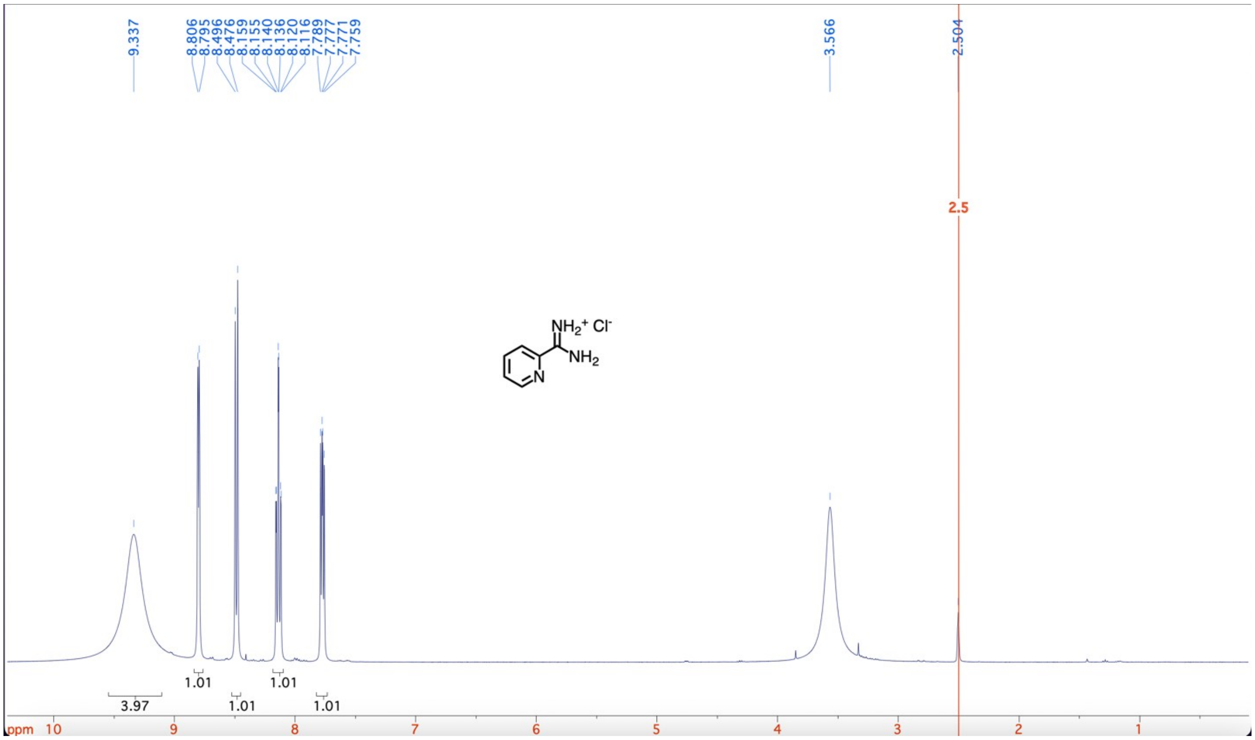

^{1}H NMR spectrum of compound **1**.

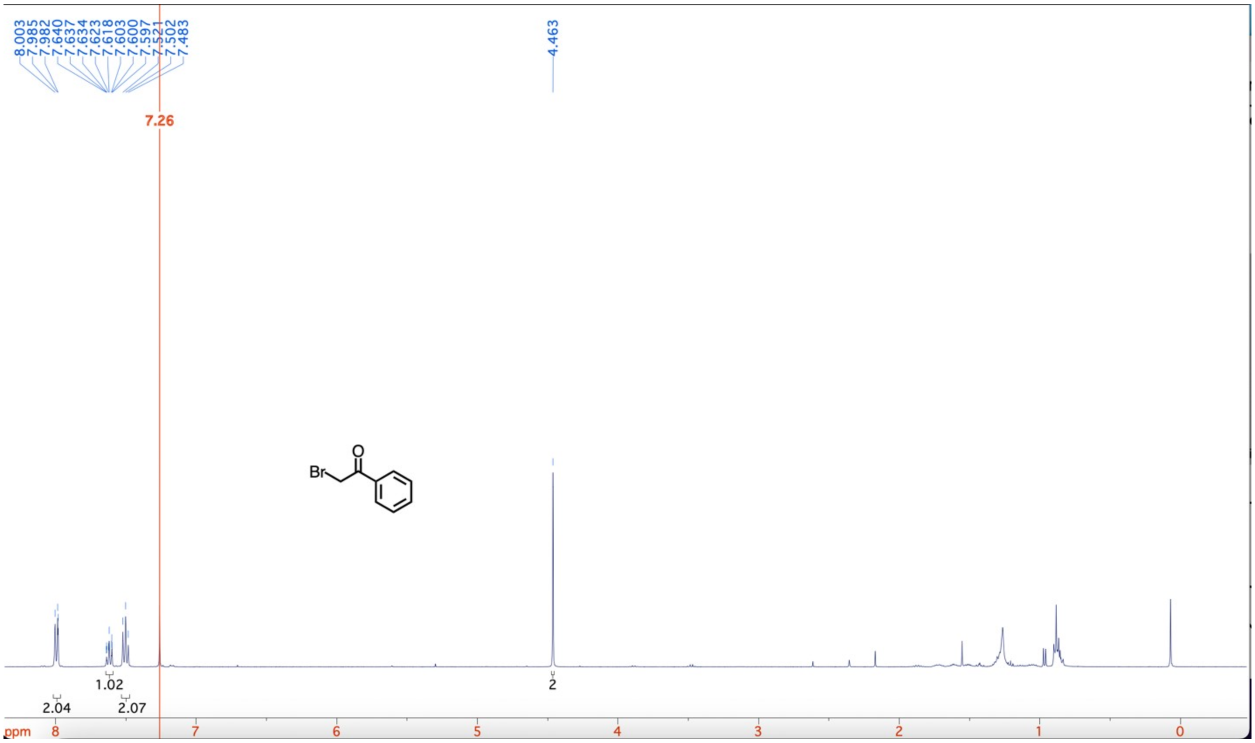

^{1}H NMR spectrum of compound **2** in $CDCl_3$.

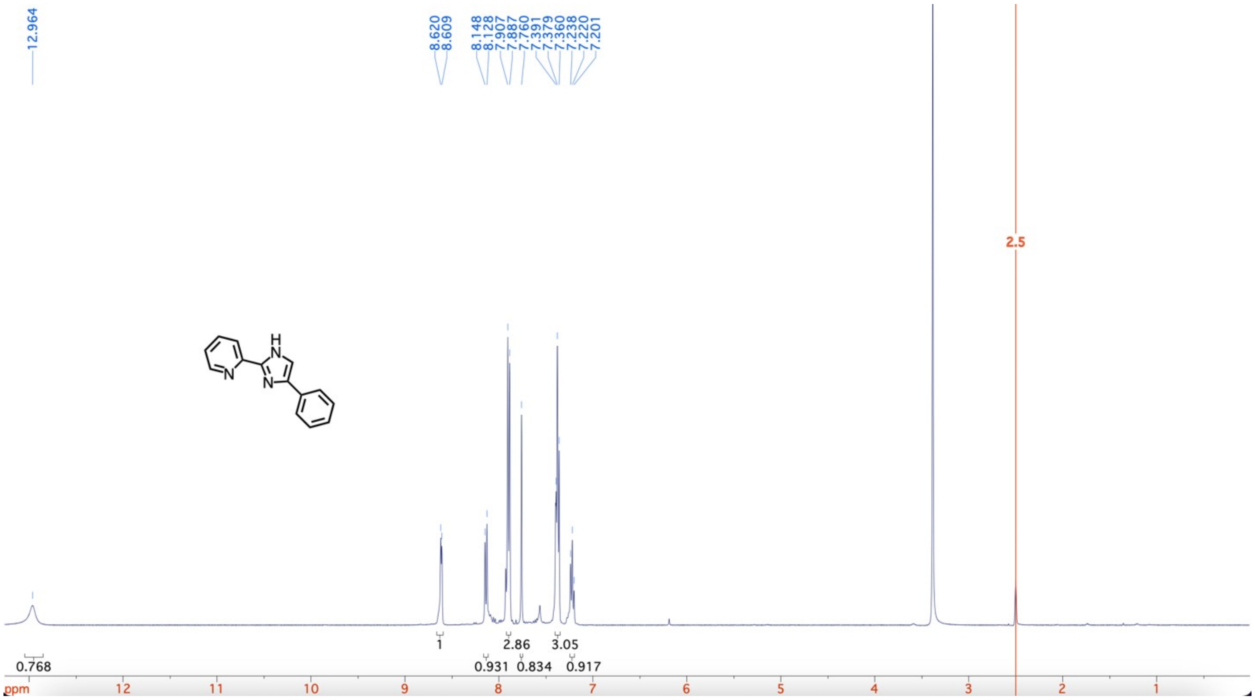

^{1}H NMR spectrum of compound **3** in d_6-DMSO.

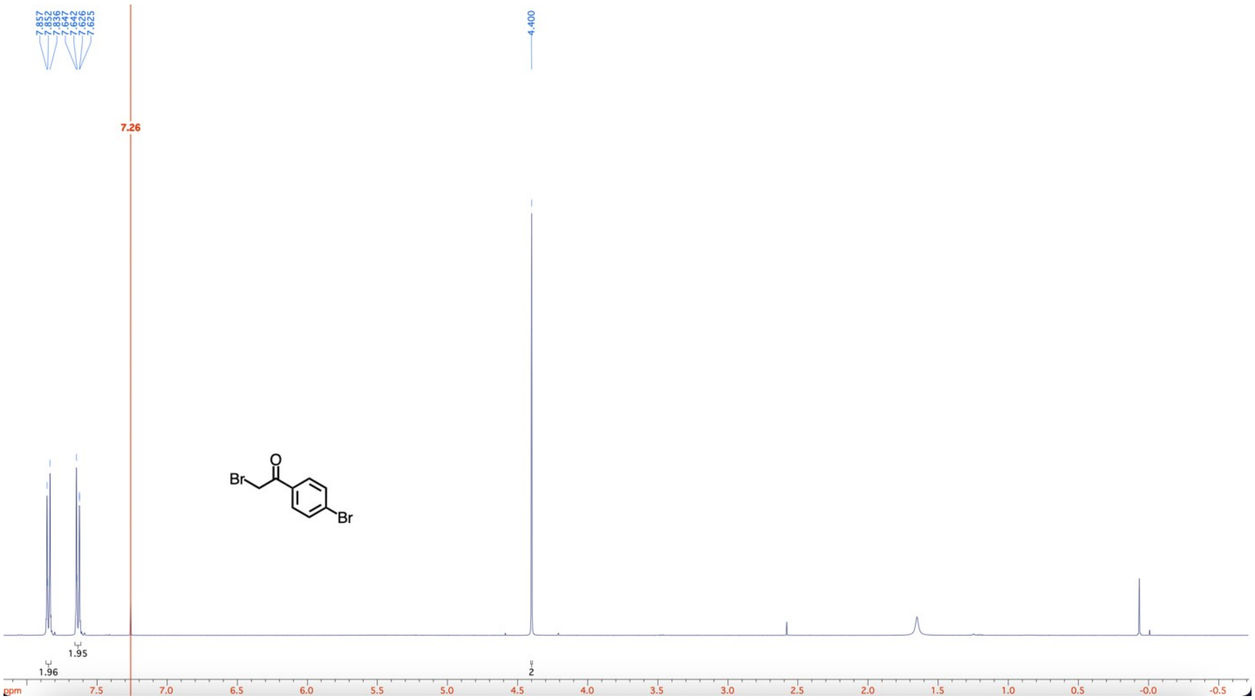

^{1}H NMR spectrum of compound **5** in $CDCl_3$.

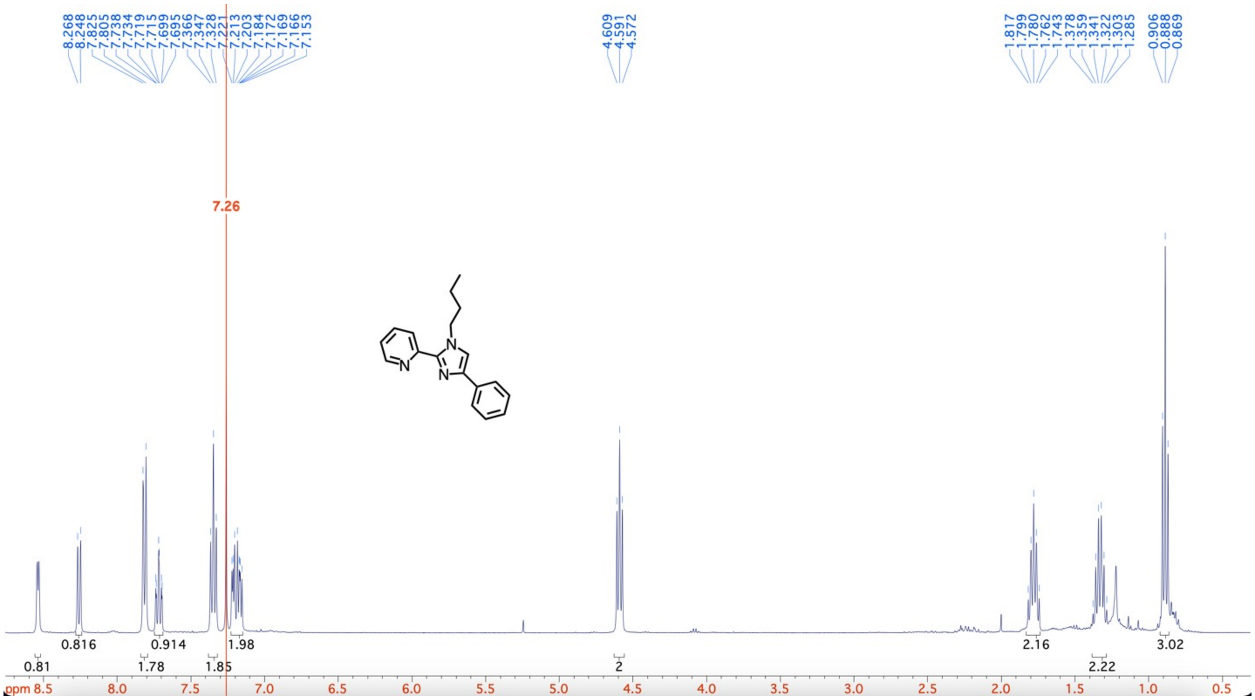

^{1}H NMR spectrum of compound **4** in $CDCl_3$.

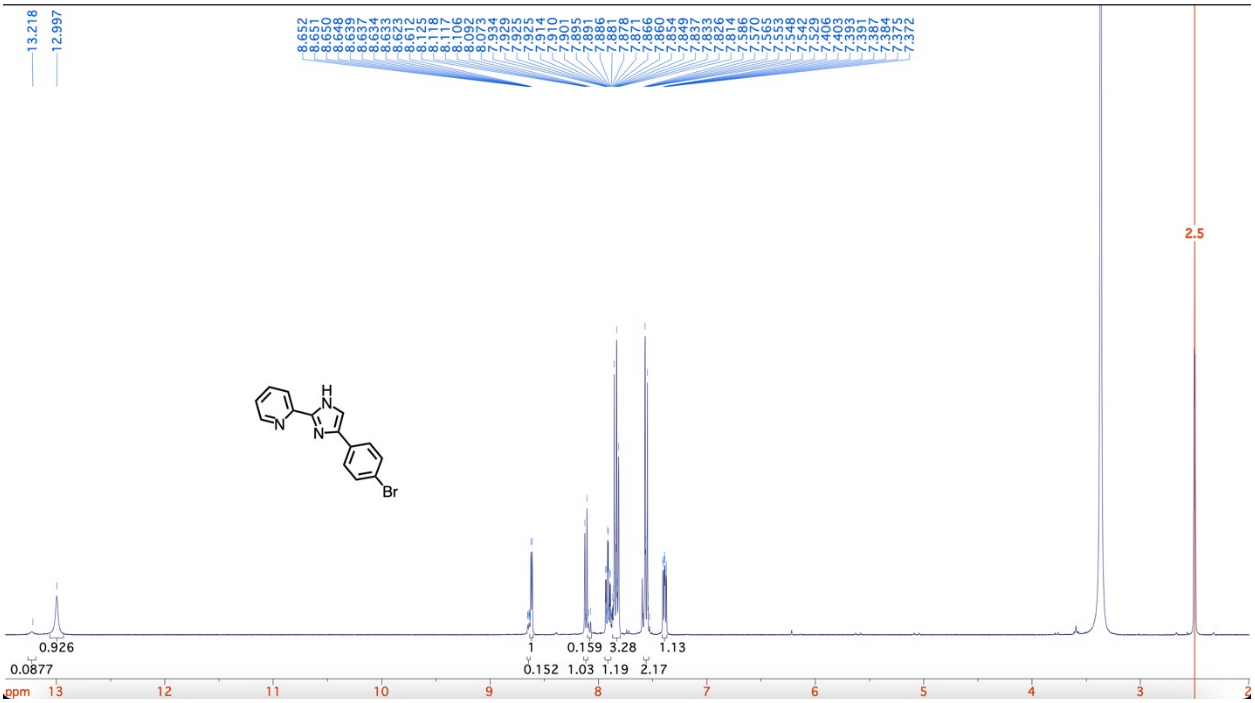

^{1}H NMR spectrum of compound **6** in d_6-DMSO.

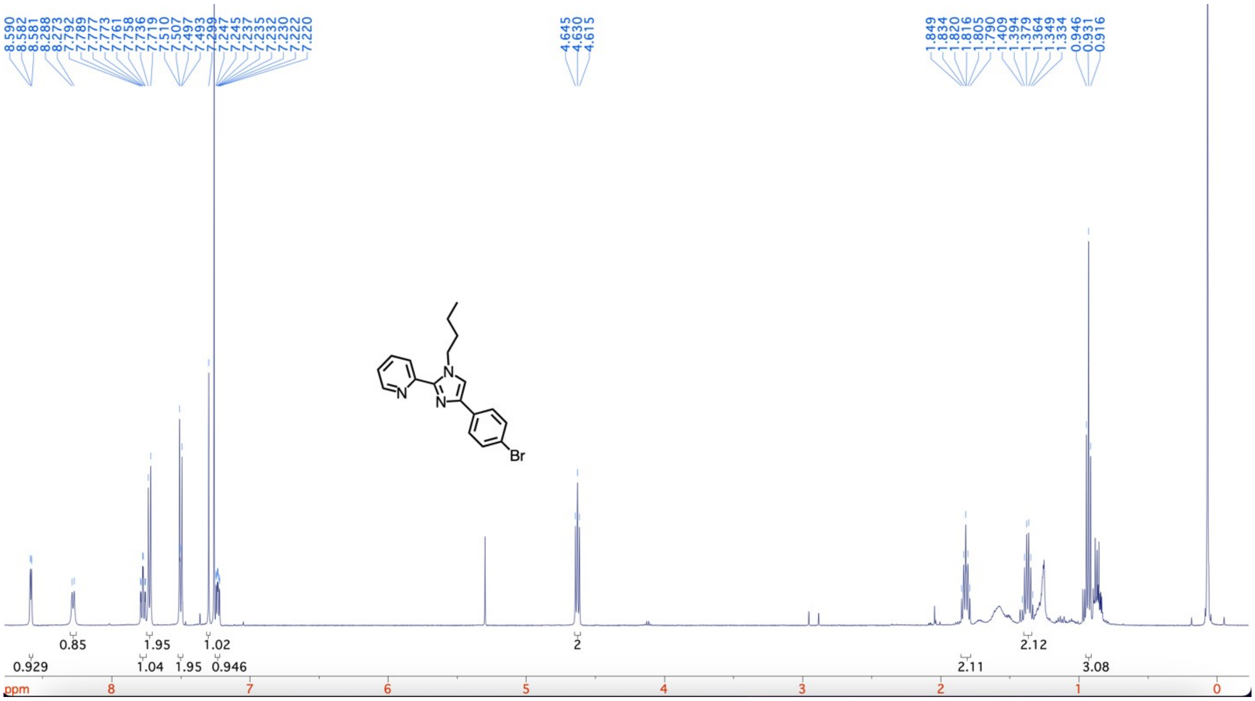

^{1}H NMR spectrum of compound **7** in $CDCl_3$.

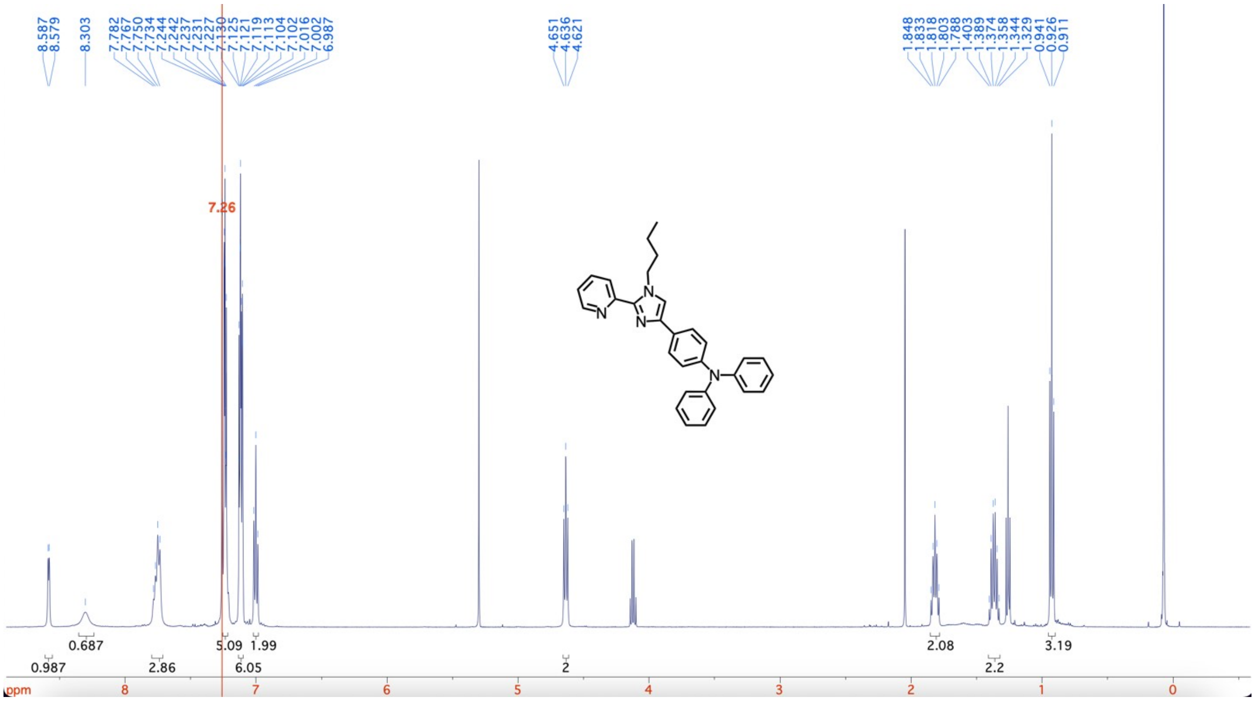

^{1}H NMR spectrum of compound **8** in $CDCl_3$.

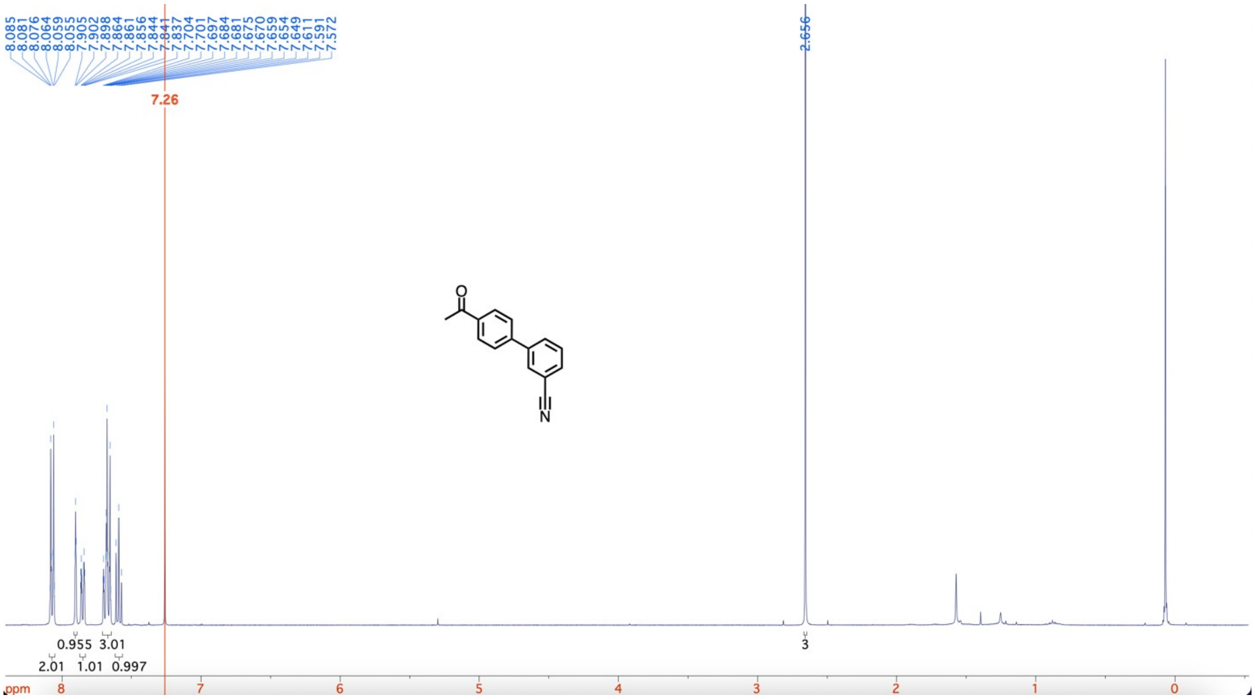

^{1}H NMR spectrum of compound **9** in $CDCl_3$.

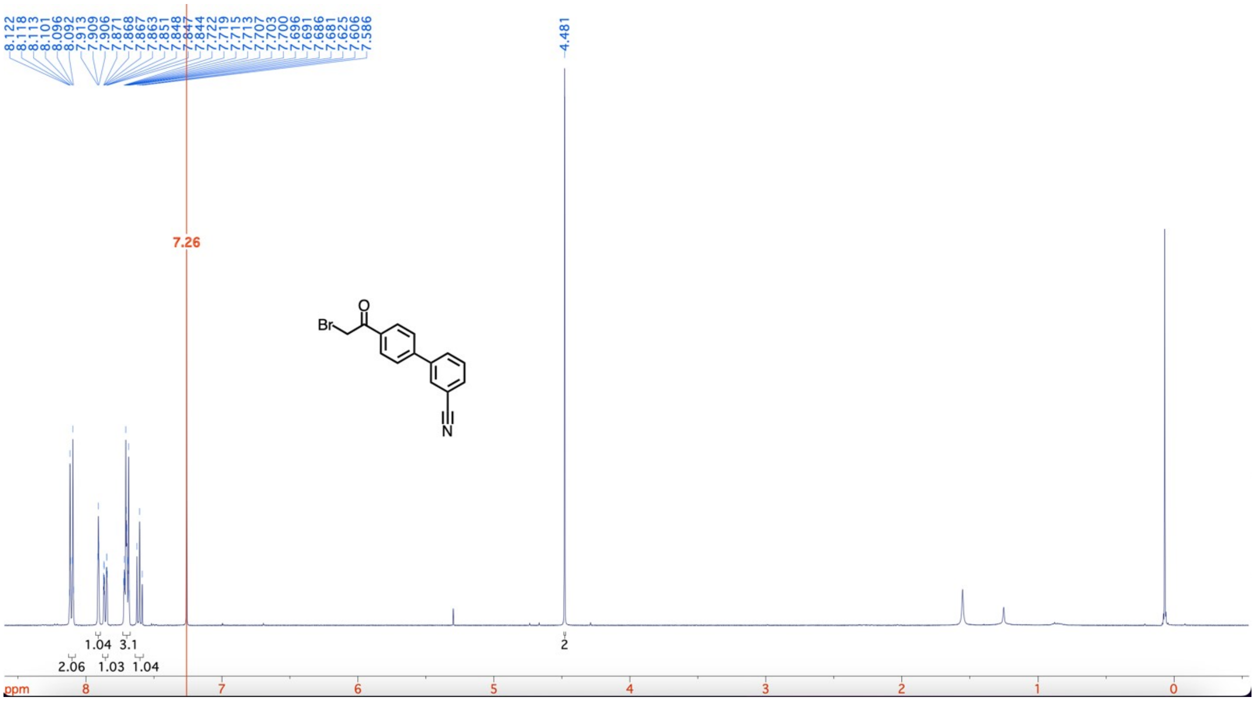

^{1}H NMR spectrum of compound **10** in $CDCl_3$.

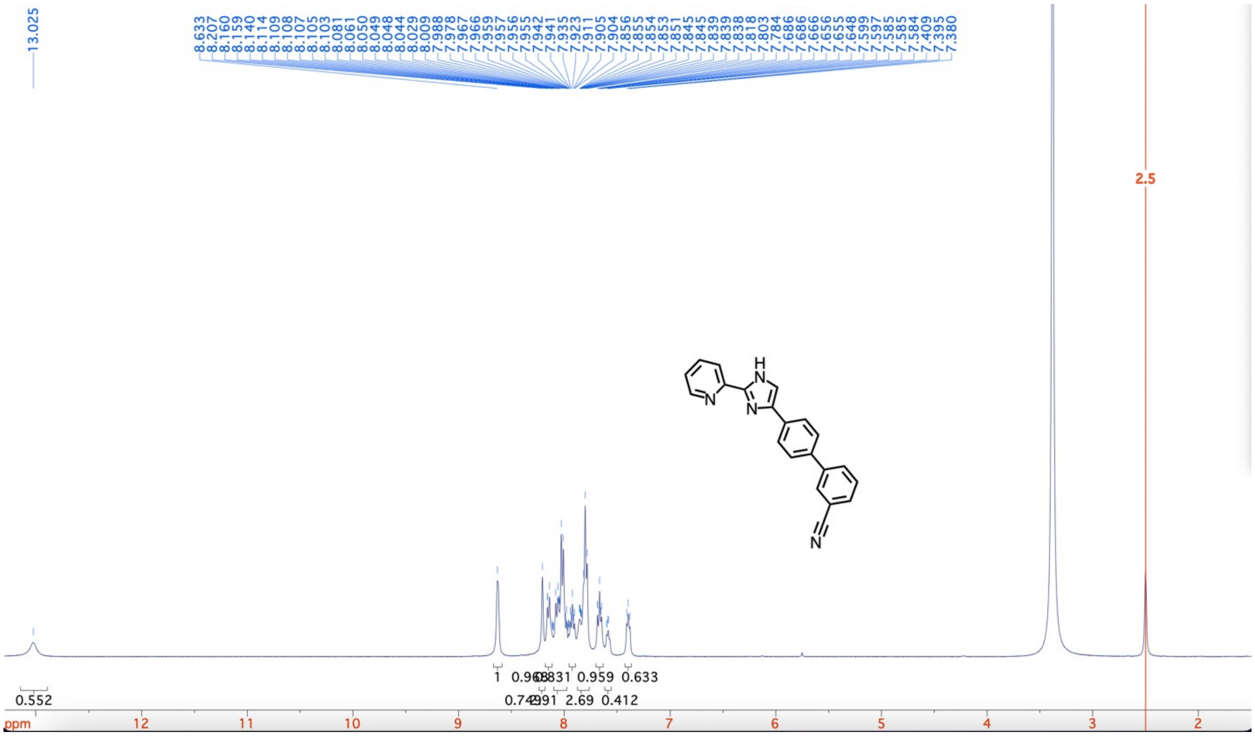

^{1}H NMR spectrum of compound **11** in d_6-DMSO.

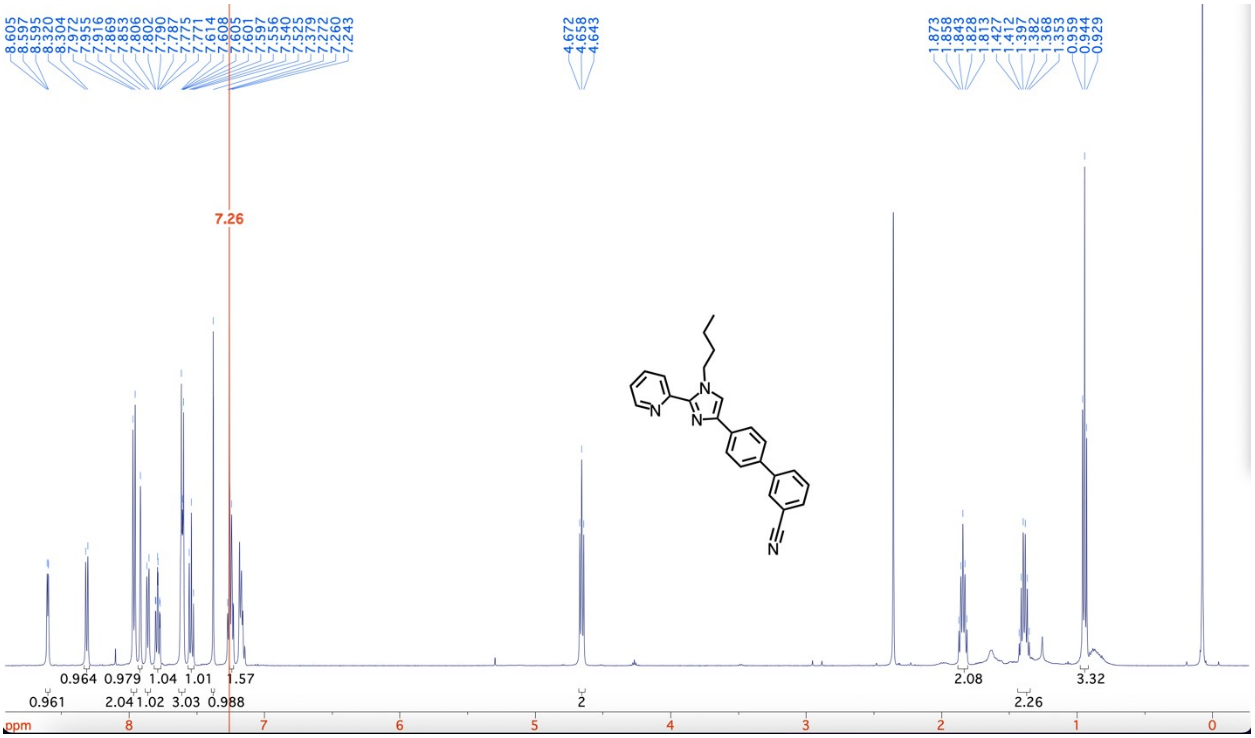

^{1}H NMR spectrum of compound **12** in $CDCl_3$.

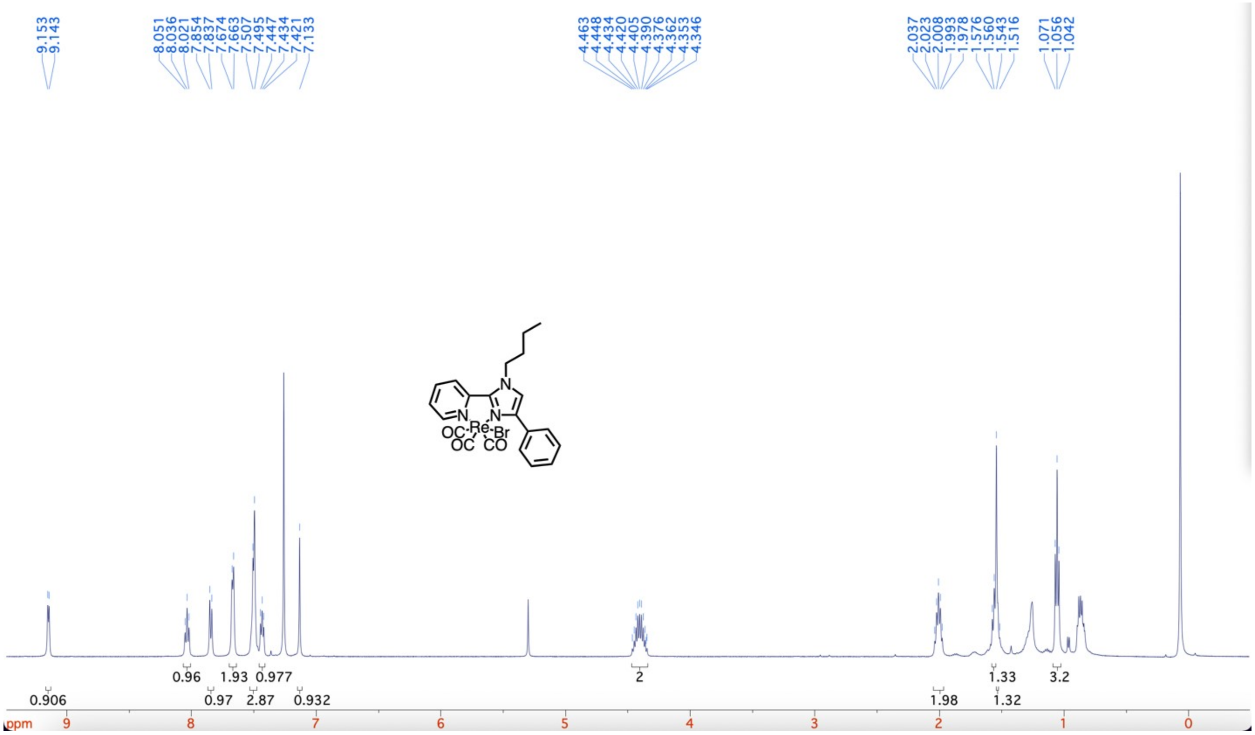

^{1}H NMR spectrum of compound **RC2** in $CDCl_3$.

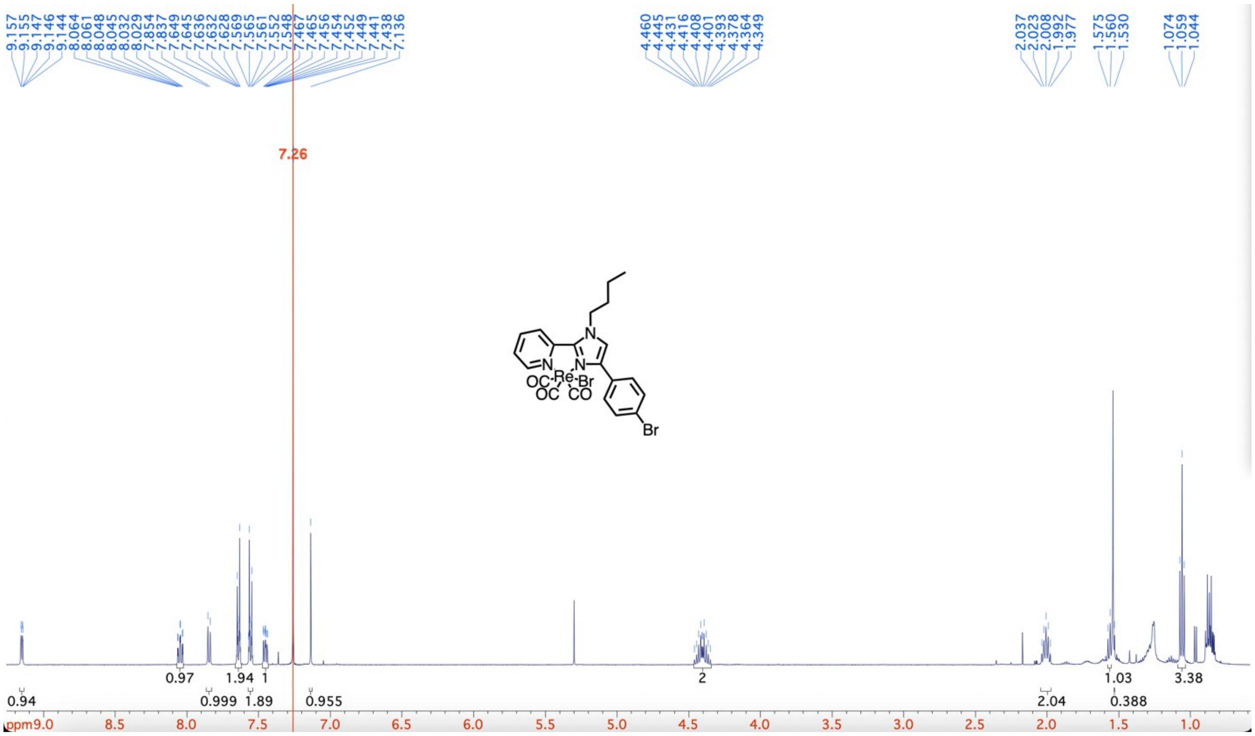

^{1}H NMR spectrum of compound **RC3** in $CDCl_3$.

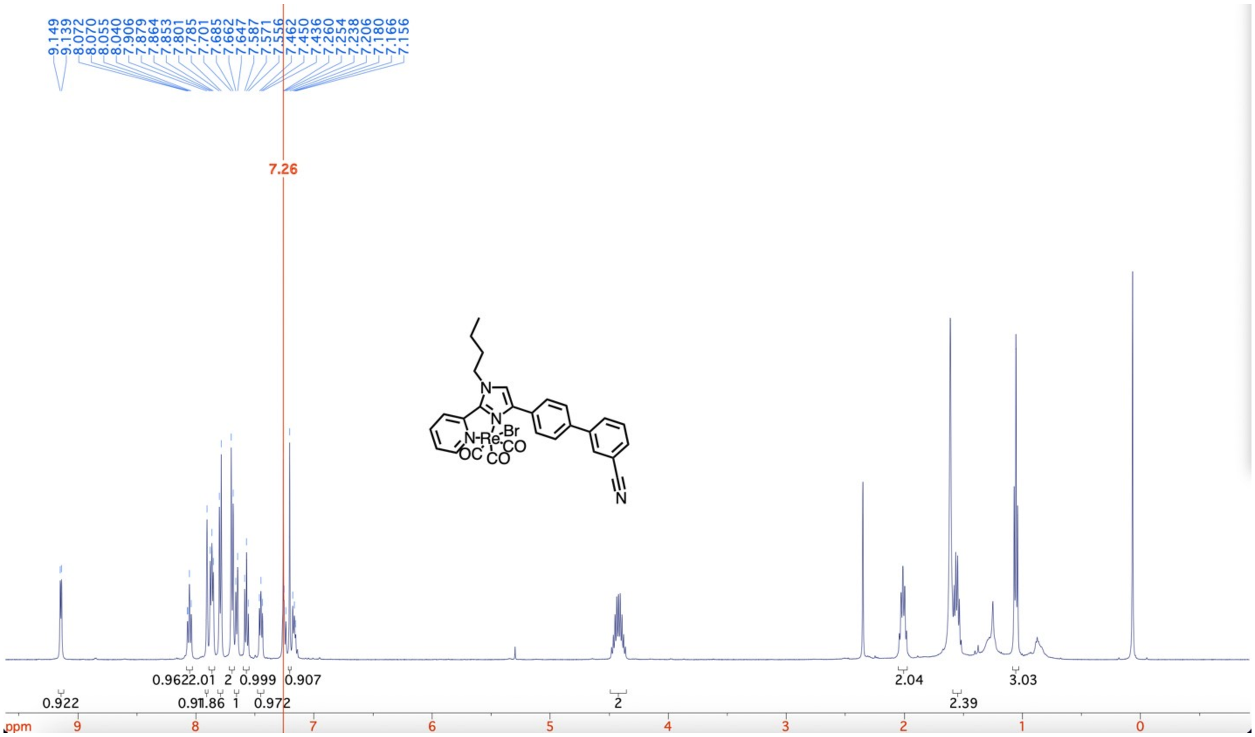

^{1}H NMR spectrum of compound **RC4** in $CDCl_3$.

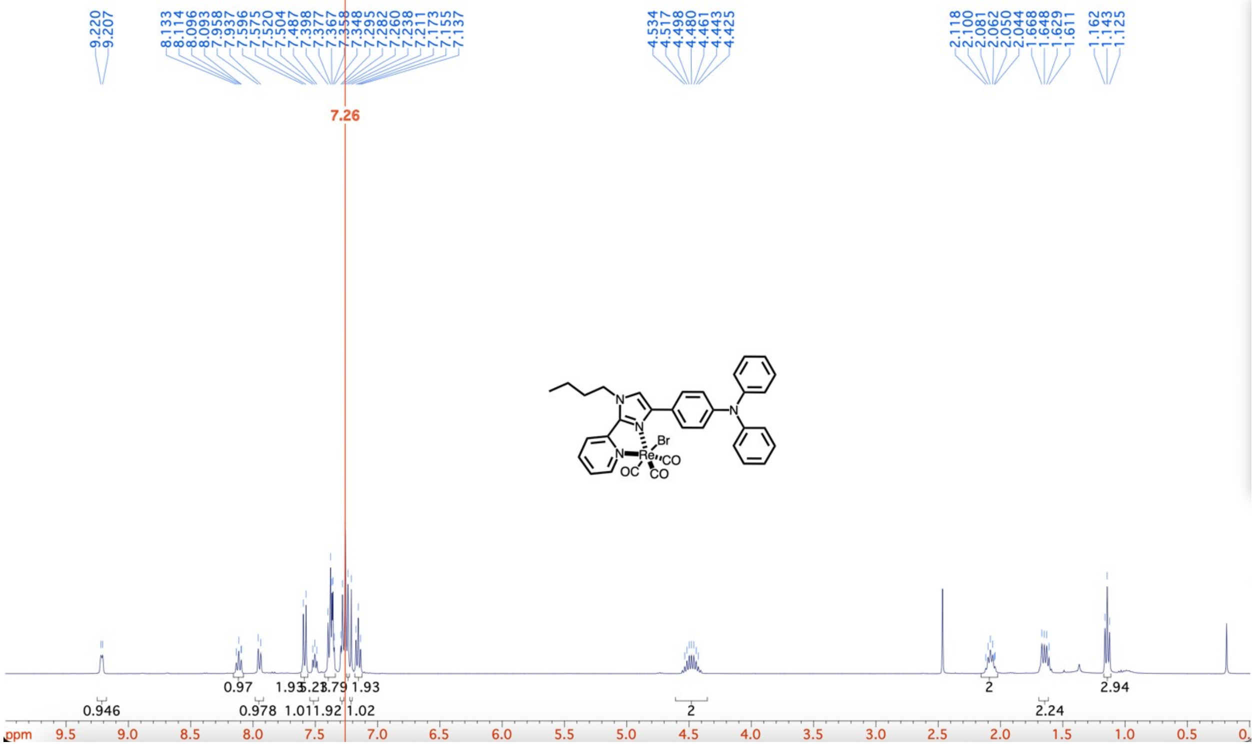

^{1}H NMR spectrum of compound **RC5** in $CDCl_3$.

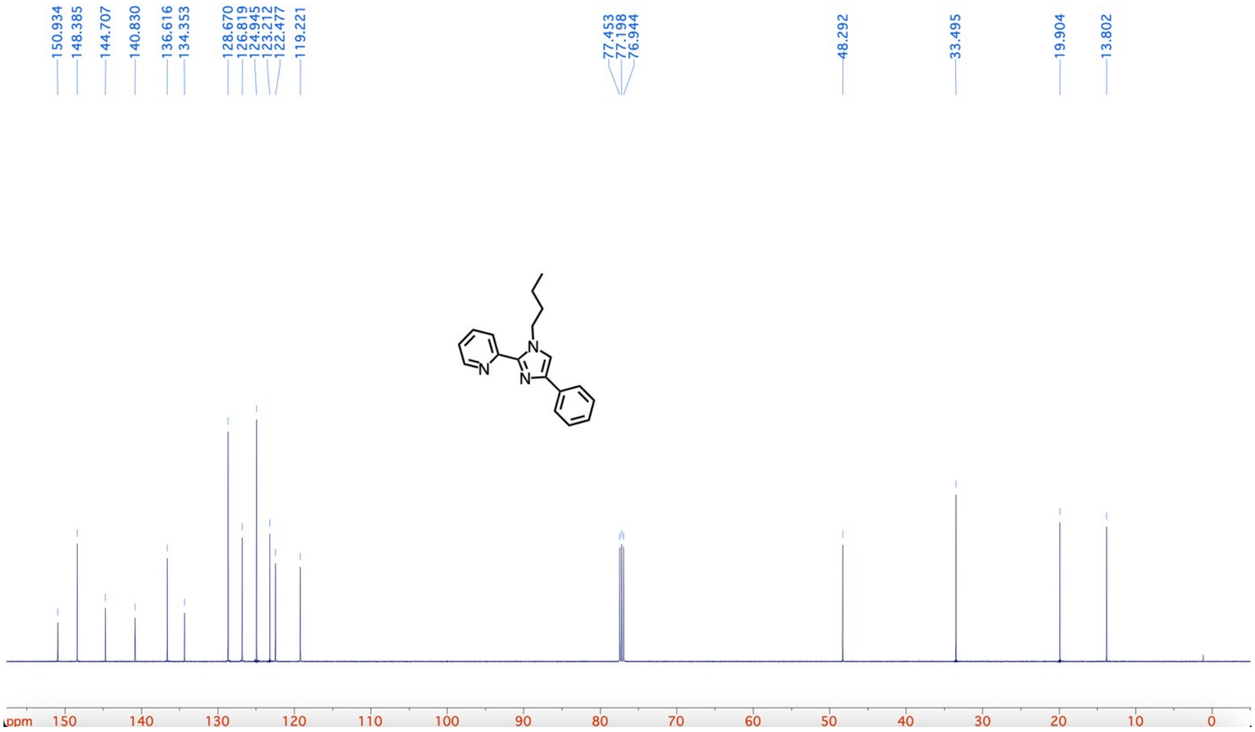

^{13}C NMR spectrum of compound **4** in $CDCl_3$.

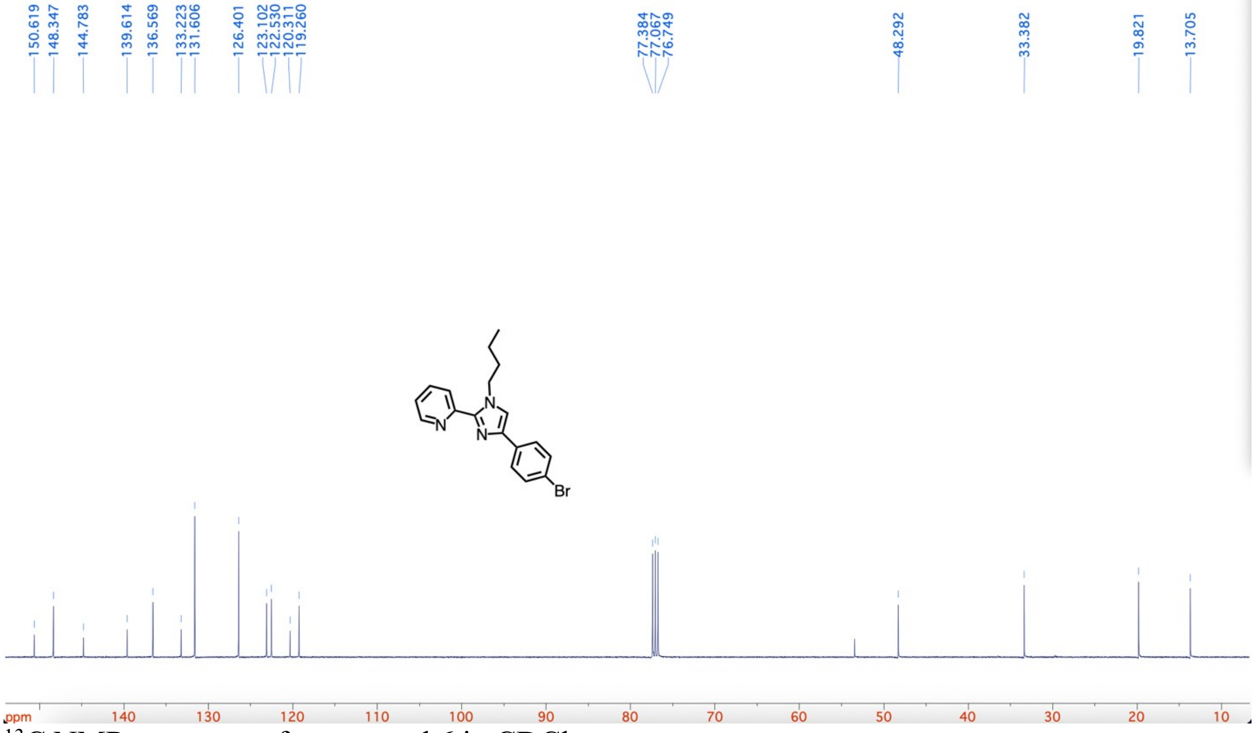

^{13}C NMR spectrum of compound **6** in $CDCl_3$.

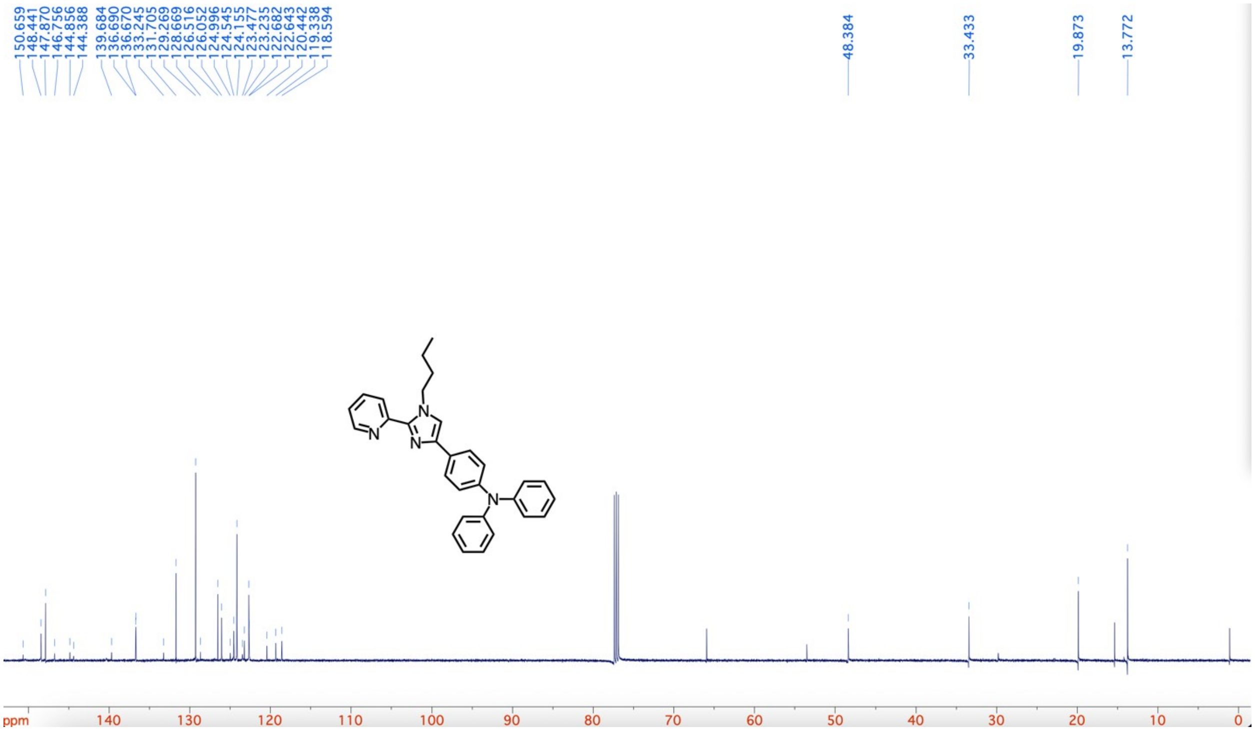
^{13}C NMR spectrum of compound **8** in $CDCl_3$.

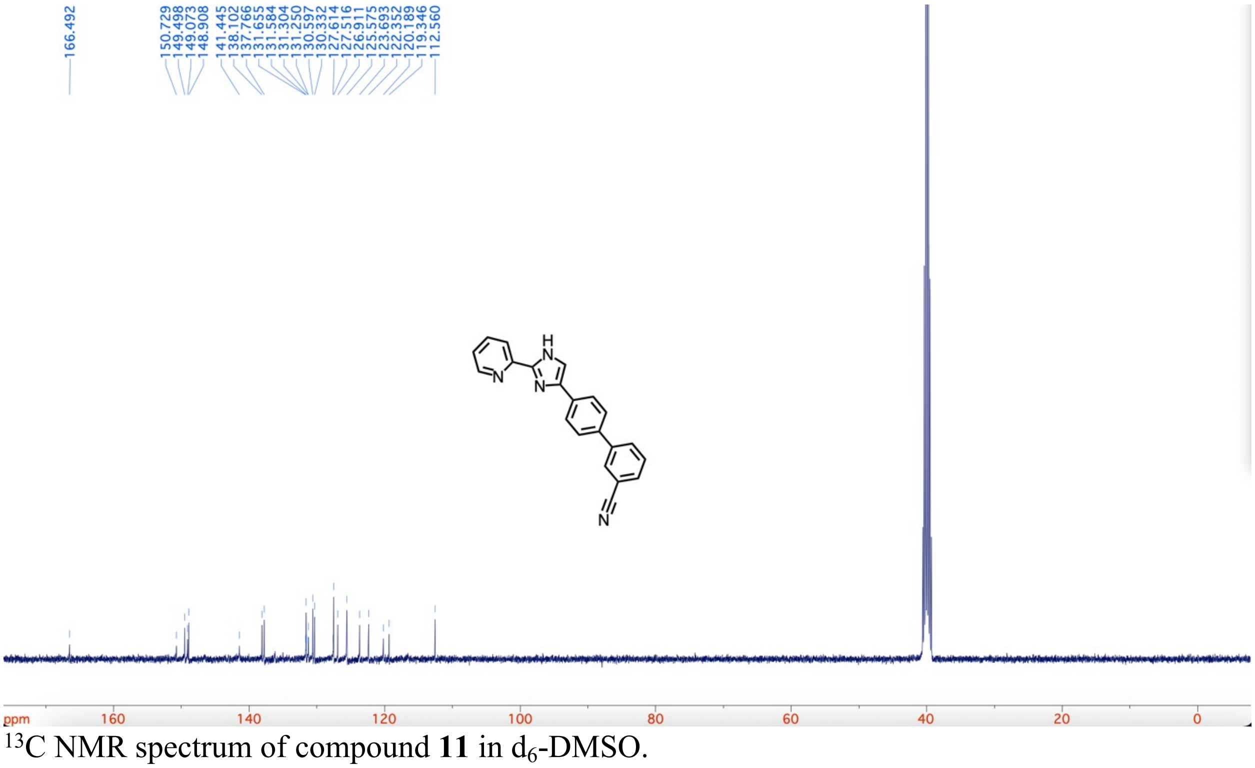
^{13}C NMR spectrum of compound **11** in d_6-DMSO.

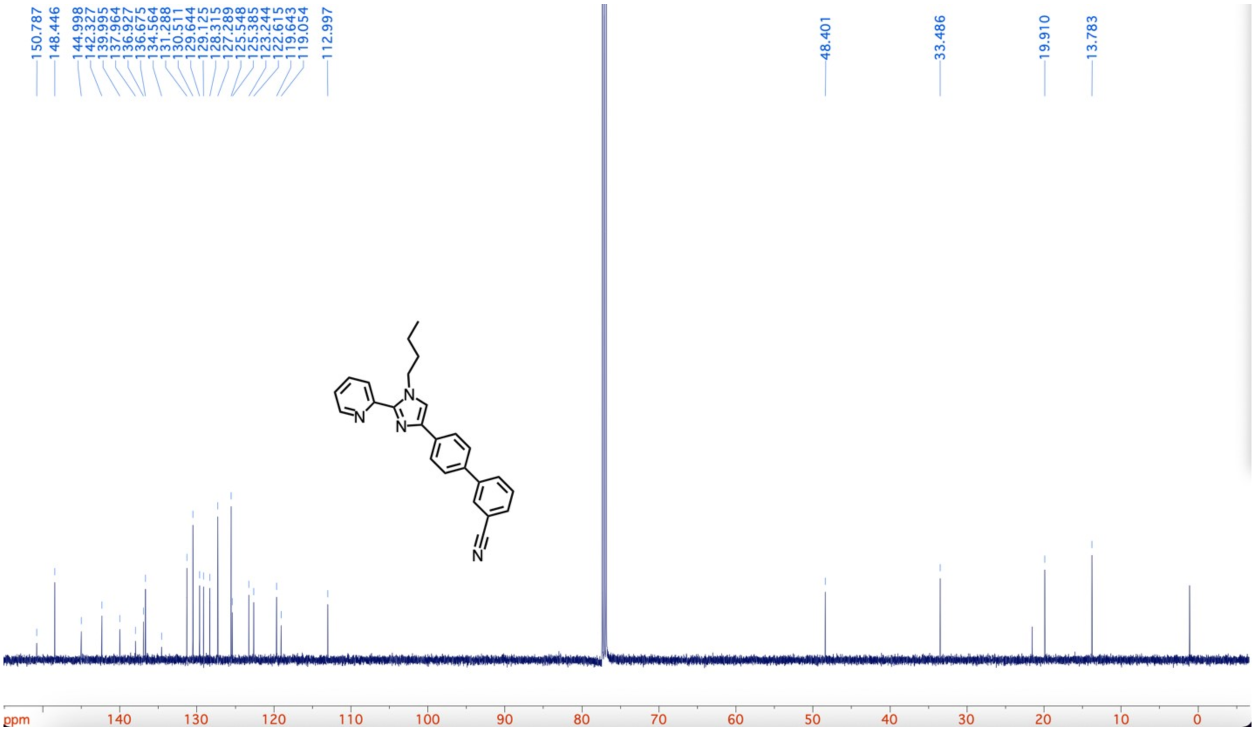

^{13}C NMR spectrum of compound **12** in $CDCl_3$.

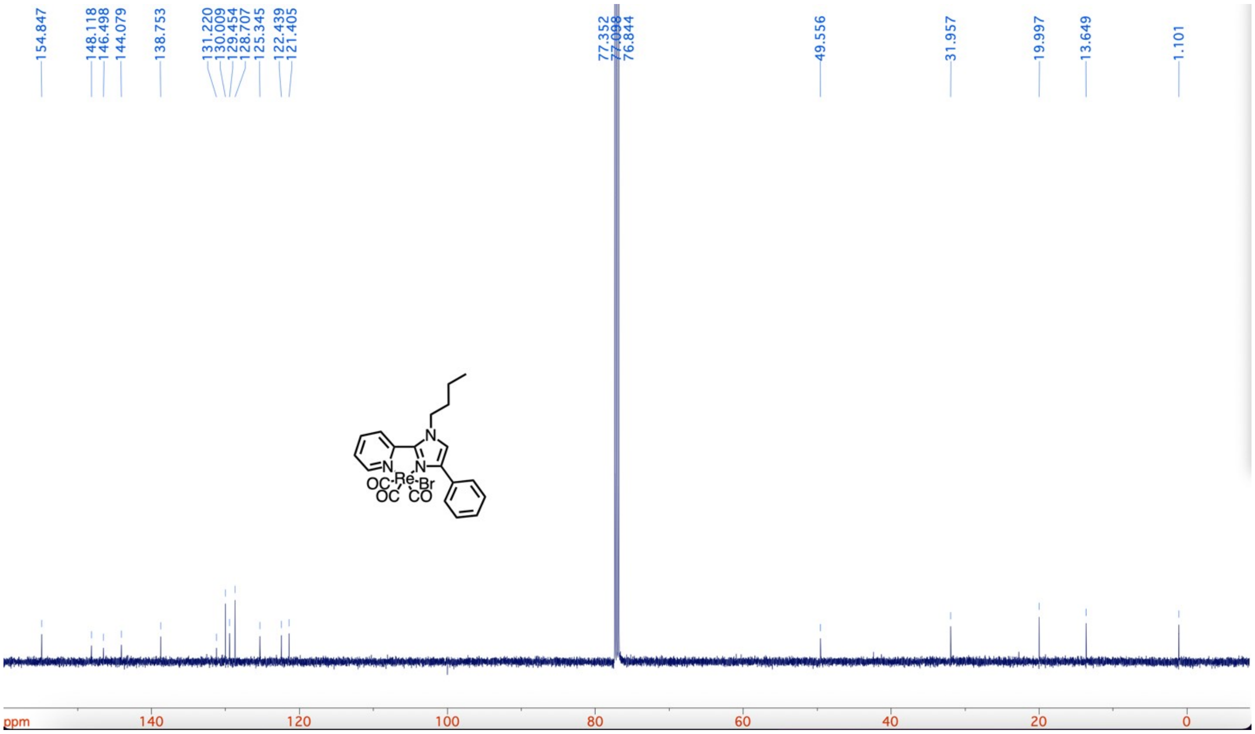

^{13}C NMR spectrum of compound **RC2** in $CDCl_3$.

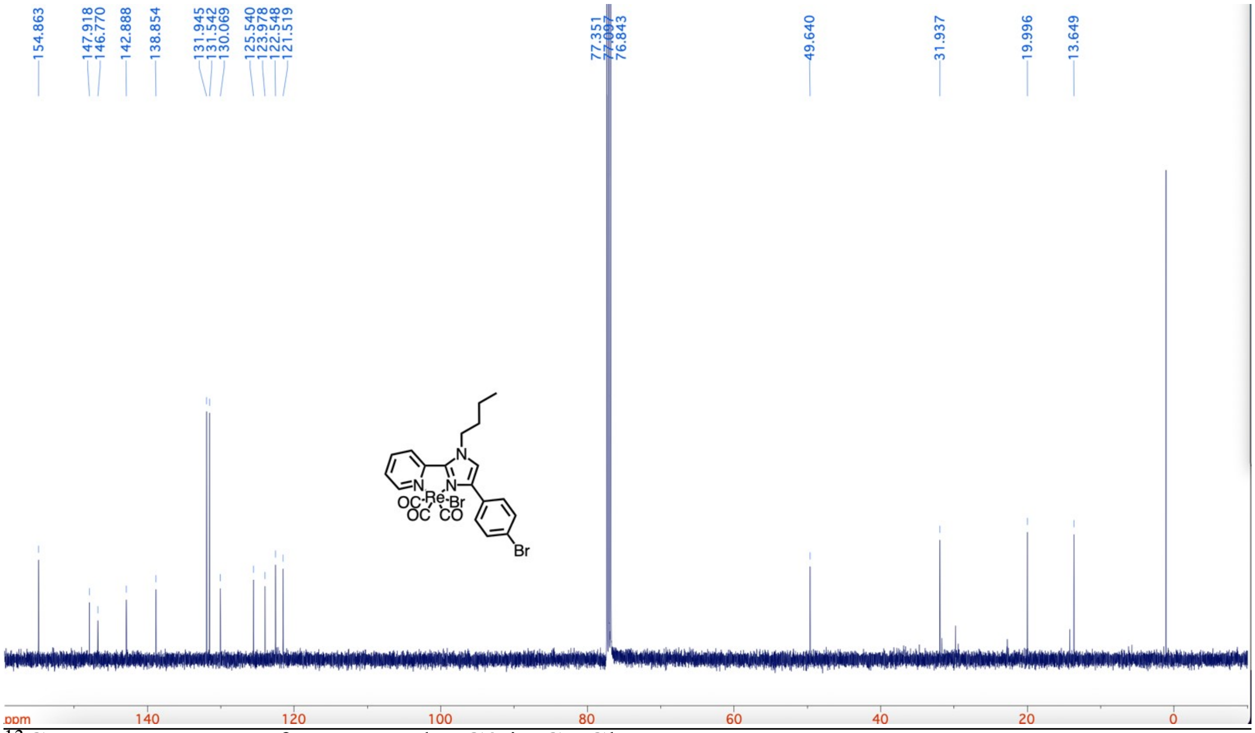

^{13}C NMR spectrum of compound **RC3** in $CDCl_3$.

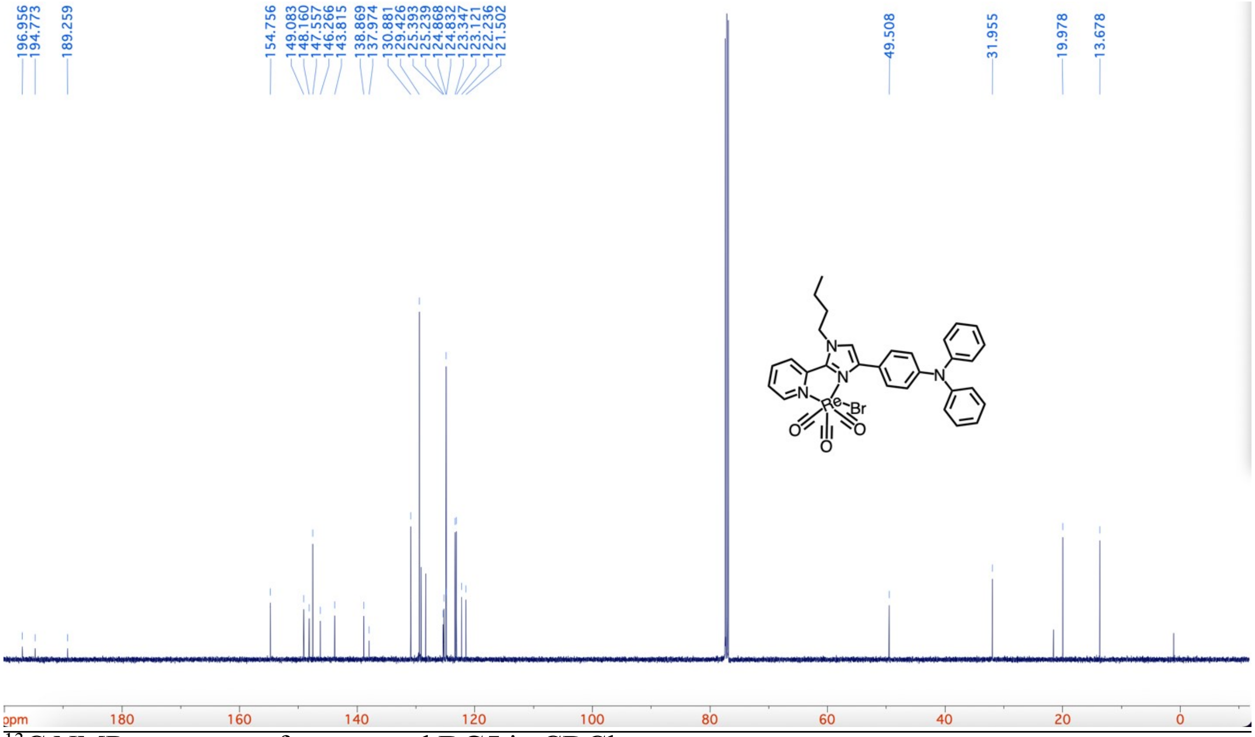

^{13}C NMR spectrum of compound **RC5** in $CDCl_3$.

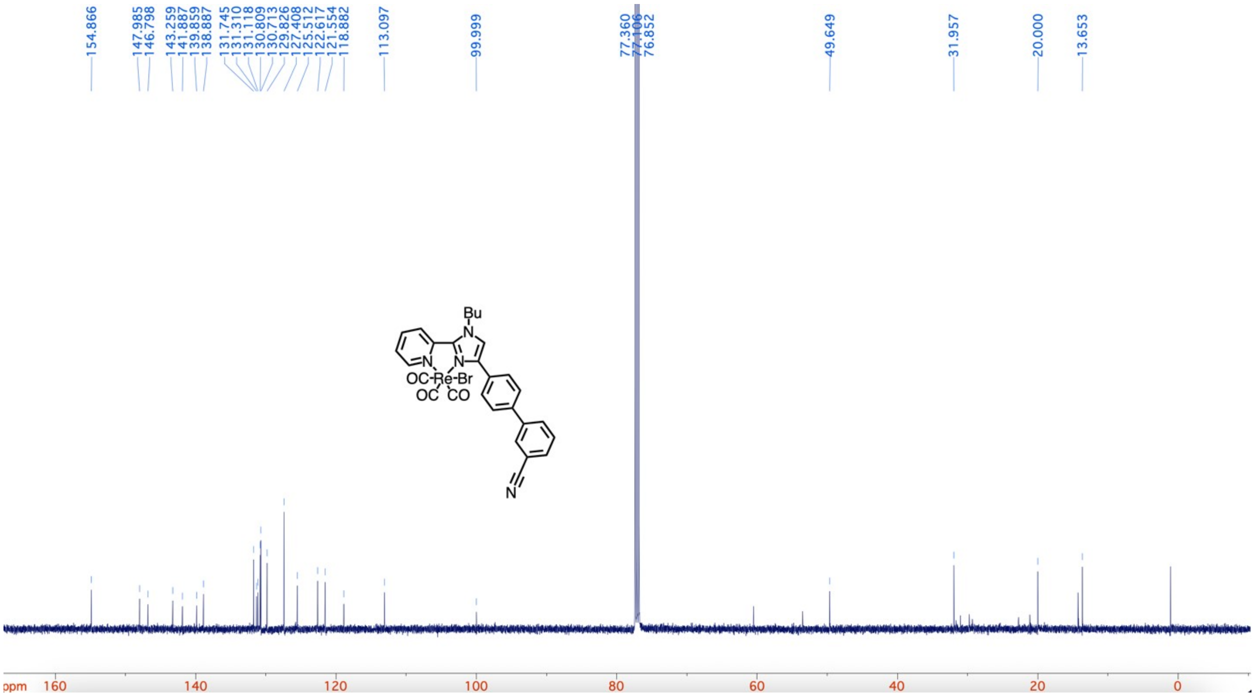

^{13}C NMR spectrum of compound **RC4** in $CDCl_3$.
